# Impaired angiogenesis in diabetic critical limb ischemia is mediated by a miR-130b/INHBA signaling axis

**DOI:** 10.1172/jci.insight.163041

**Published:** 2023-05-22

**Authors:** Henry S. Cheng, Daniel Pérez-Cremades, Rulin Zhuang, Anurag Jamaiyar, Winona Wu, Jingshu Chen, Aspasia Tzani, Lauren Stone, Jorge Plutzky, Terence E. Ryan, Philip P. Goodney, Mark A. Creager, Marc S. Sabatine, Marc P. Bonaca, Mark W. Feinberg

**Affiliations:** 1 Department of Medicine, Cardiovascular Division, Brigham and Women’s Hospital, Harvard Medical School, Boston, Massachusetts, USA.; 2 Department of Physiology, University of Valencia, and INCLIVA Biomedical Research Institute, Valencia, Spain.; 3 Department of Cardiothoracic Surgery, Nanjing Drum Tower Hospital, The Affiliated Hospital of Medical School of Nanjing University, Nanjing, China.; 4 Department of Applied Physiology and Kinesiology, University of Florida, Gainesville, Florida, USA.; 5 Heart and Vascular Center, Dartmouth-Hitchcock Medical Center and Geisel School of Medicine at Dartmouth, Lebanon, New Hampshire, USA.; 6 CPC Clinical Research, University of Colorado, Denver, Colorado, USA.

**Keywords:** Angiogenesis, Vascular Biology, Endothelial cells, Mouse models, Noncoding RNAs

## Abstract

Patients with peripheral artery disease (PAD) and diabetes compose a high-risk population for development of critical limb ischemia (CLI) and amputation, although the underlying mechanisms remain poorly understood. Comparison of dysregulated microRNAs from diabetic patients with PAD and diabetic mice with limb ischemia revealed the conserved microRNA, *miR–130b-3p*. In vitro angiogenic assays demonstrated that *miR-130b* rapidly promoted proliferation, migration, and sprouting in endothelial cells (ECs), whereas *miR-130b* inhibition exerted antiangiogenic effects. Local delivery of *miR-130b* mimics into ischemic muscles of diabetic mice (*db/db*) following femoral artery ligation (FAL) promoted revascularization by increasing angiogenesis and markedly improved limb necrosis and amputation. RNA-Seq and gene set enrichment analysis from *miR-130b*–overexpressing ECs revealed the BMP/TGF-β signaling pathway as one of the top dysregulated pathways. Accordingly, overlapping downregulated transcripts from RNA-Seq and miRNA prediction algorithms identified that *miR-130b* directly targeted and repressed the TGF-β superfamily member inhibin-β-A (*INHBA*). miR-130b overexpression or siRNA-mediated knockdown of INHBA induced IL-8 expression, a potent angiogenic chemokine. Lastly, ectopic delivery of silencer RNAs (siRNA) targeting *Inhba* in *db/db* ischemic muscles following FAL improved revascularization and limb necrosis, recapitulating the phenotype of *miR-130b* delivery. Taken together, a *miR-130b*/*INHBA* signaling axis may provide therapeutic targets for patients with PAD and diabetes at risk of developing CLI.

## Graphical abstract

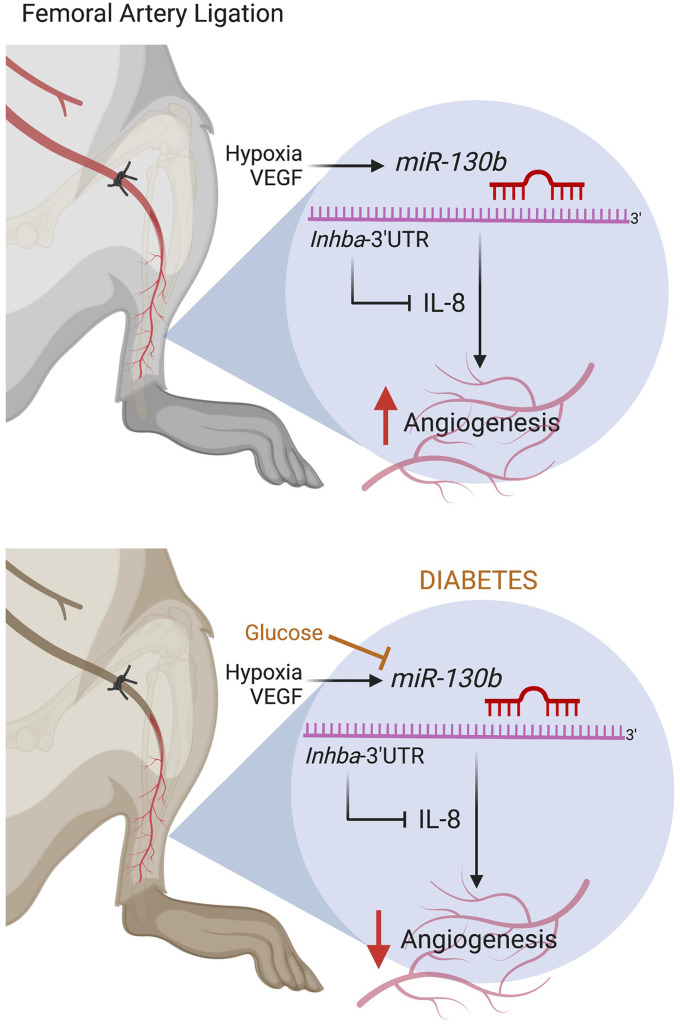

## Introduction

Angiogenesis, the growth of new blood vessels from preexisting vessels, is a physiologic process that is vital for maintenance of tissues. When angiogenesis is impaired, tissues become ischemic, and pathologies emerge such as peripheral artery disease (PAD). Approximately 200 million people worldwide are affected by PAD, and a subset of 1%–2% go on to develop critical limb ischemia (CLI) (1). Many risk factors contribute to the manifestation of CLI, including smoking, age, hypertension, and diabetes mellitus (DM) (2). In fact, patients with PAD and DM are at 20%–30% higher risk of developing cardiovascular and limb events compared with non-DM patients (3). Hyperglycemia or insulin resistance are known to negatively impact angiogenesis and a wide range of endothelial cell (EC) functions (4). Impaired angiogenesis leads to worse outcomes in mouse diabetic hindlimb ischemia models (5) and in patients with diabetes (6). However, delivery of recombinant growth factors such as vascular endothelial growth factor A (VEGF-A) offered no therapeutic benefits in clinical trials for PAD, suggesting that regulation of angiogenesis goes beyond ligand-receptor interactions (7).

MicroRNAs (miRNAs) are small, single-stranded noncoding RNAs capable of mediating posttranscriptional repression of mRNA transcripts. miRNAs are also capable of targeting multiple mRNAs that often converge along similar signaling pathways, thereby amplifying biological responses (8). Therefore, understanding the targets of certain miRNAs can, in turn, reveal important pathway networks in disease pathogenesis. In addition, emerging studies highlight that evolutionary conserved miRNAs may be particularly effective for uncovering stage-specific disease states (6, 9). Since diabetes is a major contributor in the development of CLI, focused studies that explore miRNAs conserved in both patients and mouse models of diabetes and PAD hold great promise to uncover insights and signaling pathways relevant for this aggressive disease.

Herein, we evaluate the role of *miR-130b* selected from a discovery platform of dysregulated miRNAs in patients with diabetes who have PAD and mice with experimental PAD that have diabetes. We found that *miR-130b* promotes angiogenesis in ECs and rapidly facilitates revascularization in a diabetic murine model of hindlimb ischemia. Furthermore, we identified Inhibin subunit β A (INHBA), a member of the TGF-β superfamily of proteins, to be a direct target of *miR-130b* and to contribute to regulating angiogenesis in diabetic mice.

## Results

### Identification of miR–130b-3p in experimental PAD in diabetic mice and in patients with PAD and diabetes.

Plasma from patients with PAD and diabetes were used for miRNA-Seq. Samples were separated into 2 groups based on patient Fontaine classifications as a metric of severity of disease. Fontaine class I and II (FI/II) include patients who are typically asymptomatic or with intermittent claudication (IC), whereas FIII/IV include patients who are at higher risk of developing CLI, adverse limb events, and cardiovascular death (10). Analysis was performed comparing the high-risk FIII/IV with the low-risk FI/II group, resulting in 209 downregulated and 188 upregulated miRNAs (adjusted *P* [*P*_adj_] < 0.05, fold change > 1.5).

**Figure 1:**
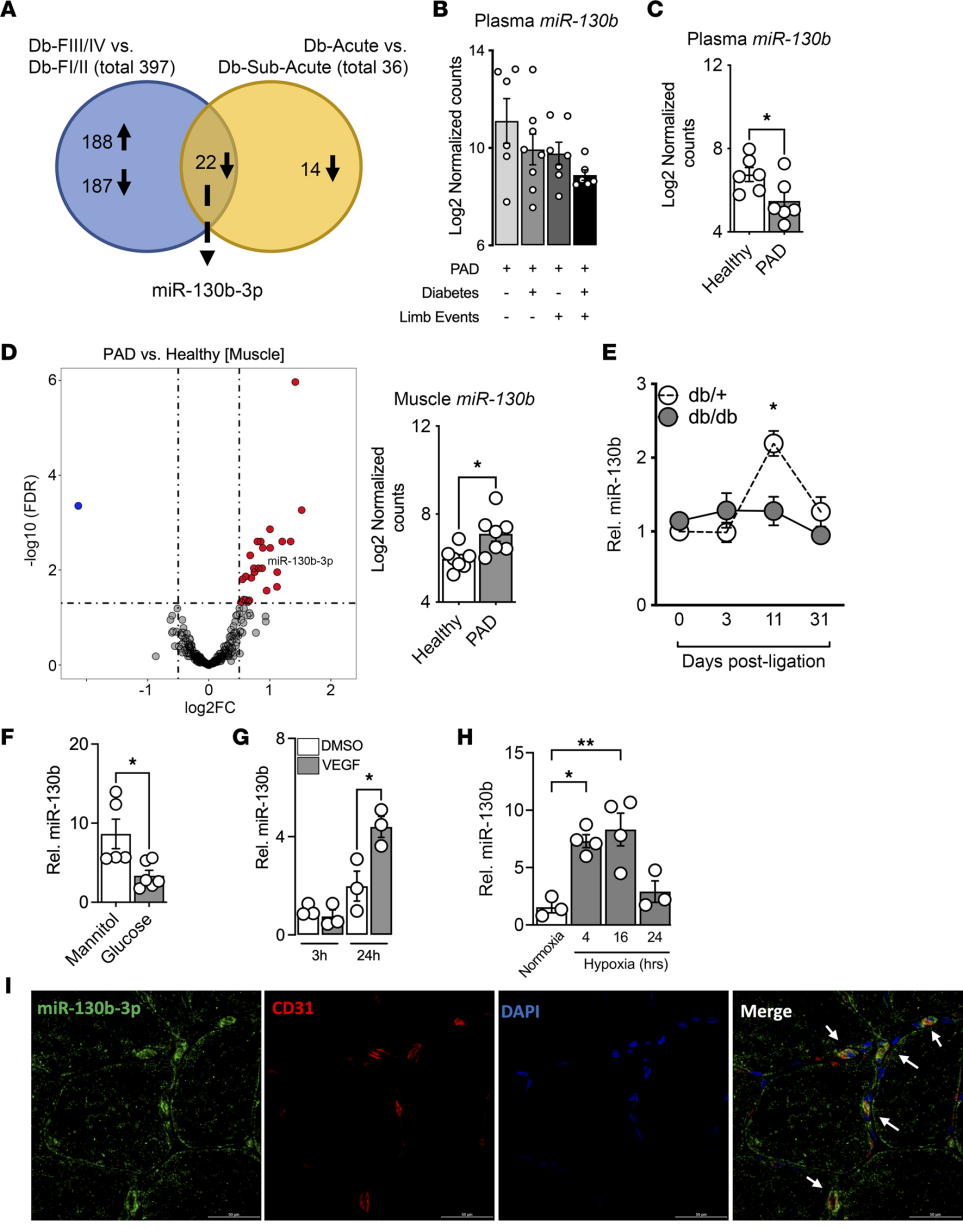
Identification of *miR–130b-3p* in experimental PAD in diabetic mice and in patients with PAD and diabetes. (**A**) Venn diagram indicating number of miRNAs commonly dysregulated from human and mouse miRNA-Seq analysis (F, Fontaine; Db, diabetes). (**B**) Normalized counts of *miR-130b* in patients with PAD and with or without diabetes or limb events (*n* = 6–7). (**C**) Normalized counts of *miR-130b* in plasma from a separate cohort of patients with PAD compared with plasma from healthy individuals (*n* = 6). (**D**) (Left) Volcano plot highlighting *miR-130b* in muscles from patients with PAD compared with healthy individuals. (Right) Normalized counts of *miR-130b* in muscles of patients with PAD compared with healthy individuals (*n* = 7). (**E**) *miR-130b* expression normalized to *U6* in ischemic gastrocnemius of *db/+* and *db/db* mice at different time points after FAL. Comparison between groups at specific time points by unpaired Student’s *t* test (d0, *n* = 11–12; d3, *n* = 7–12; d11, *n* = 5–6; d31, *n* = 4–7). (**F**–**H**) Relative expression of *miR-130b* normalized to *U6* in HUVECs under different conditions: after 72 hours of D-glucose compared with mannitol control (*n* = 5–6) (**F**); after 3 or 24 hours of VEGF-A stimulation (*n* = 3) (**G**); or after 4, 16, and 24 hours of 2% hypoxia compared with normoxia control (*n* = 3-4) (**H**), performed with 2-way ANOVA. (**I**) Representative immunofluorescence images of human skeletal muscles from patients with diabetes and PAD stained for *miR–130b-3p* (green), CD31 (red), DAPI (blue), and merged, with arrows indicating colocalization. Scale bar: 50 μm. Statistics performed using unpaired 2-tailed Student’s *t* test unless stated otherwise. *P < 0.05, **P < 0.01.

To mirror human PAD and CLI in mice, we performed 2 different types of surgeries on the femoral artery: (a) subacute ischemia induced by insertion of 2 ameroid constrictors around the vessel (11) or (b) acute ischemia induced by ligation and cauterization. When applied in diabetic mice (*db/db*), femoral artery ligation (FAL) results in a higher frequency of ischemic limb necrosis, whereas ameroid constrictor implantation rarely triggers limb necrosis (12, 13). Hence, to identify conserved miRNAs across mice and patients, we aligned plasma miRNA-Seq from acute ischemia (i.e., FAL) in diabetic *db/db* mice to diabetic patients with FIII/IV and subacute ischemia (i.e., ameroid constrictors) in *db/db* mice to diabetic patients with FI/II. Similar to our human data set selection criteria (*P*_adj_ < 0.05, fold change > 1.5), an analysis comparing acute to subacute ischemia in *db/db* mice resulted in 36 downregulated miRNAs. Overlap of both the human and mouse miRNA-Seq analysis revealed 22 commonly dysregulated miRNAs (Figure 1A).

Within the 22 identified miRNAs, *miR-130b* followed a decreased nonstatistical trend in expression as burden of disease increased for patients with PAD (Figure 1B). In support of our findings, a miRNA-Seq data set comparing health individuals with nondiabetic patients with PAD also revealed a decrease in *miR-130b* (Figure 1C). Interestingly, skeletal muscle biopsy samples from these patients with PAD revealed the opposite, with an increased expression of *miR-130b* compared with healthy muscles (Figure 1D). This increased *miR-130b* expression was also observed in the ischemic gastrocnemius muscle in our nondiabetic (*db/+*) murine acute ischemia model at 11 days after FAL, a period when angiogenesis occurs (Figure 1E). This was not observed in *db/db* ischemic tissues, suggesting that diabetes may block the increased expression of *miR-130b*. To this end, we subjected human umbilical vein ECs (HUVECs) to D-glucose for 72 hours and found that *miR-130b* was repressed compared with mannitol-treated (osmotic control) ECs (Figure 1F). In contrast, ECs treated with VEGF induced *miR-130b* expression at a late time point (24 hours) but not early (3 hours) (Figure 1G). Furthermore, *miR-130b* is induced by 2% hypoxia at 4 and 16 hours compared with normoxia-treated ECs (Figure 1H). Lastly, we performed immunofluorescence analysis on skeletal muscle biopsies from patients with diabetes and PAD (Figure 1I). We found *miR–130b-3p* to be highly expressed, with abundance colocalized with CD31^+^ ECs.

*miR-130b* belongs to the *miR-130/301* family comprising *miR-130a*, *miR-130b*, *miR-301a*, and *miR-301b*. In our murine experimental PAD model, we found all 4 members to be induced in whole gastrocnemius tissue only in the *db/+* mice following FAL, albeit at different time points, with *miR-130a/b* expression peaking at day 11 and *miR-301a/b* at day 3 (Supplemental Figure 1; supplemental material available online with this article; https://doi.org/10.1172/jci.insight.163041DS1). Next, we performed FAL on *db/+* mice, followed by separating the EC and non-EC fractions for expression analysis. Interestingly, only *miR-130b* was induced in ECs, whereas the other family members are prominent in non-ECs (Supplemental Figure 2). Furthermore, we assessed the abundance of *miR-130b* in several different cell lines by quantitative PCR (qPCR) and found *miR-130b* expression to be more enriched in ECs compared with other cell types (Supplemental Figure 3). Collectively, we have identified *miR-130b* in diabetic mice and humans burdened with PAD/CLI, with expression kinetics associated with ECs and angiogenesis.

### Endothelial miR-130b promotes angiogenesis, migration, and proliferation.

**Figure 2:**
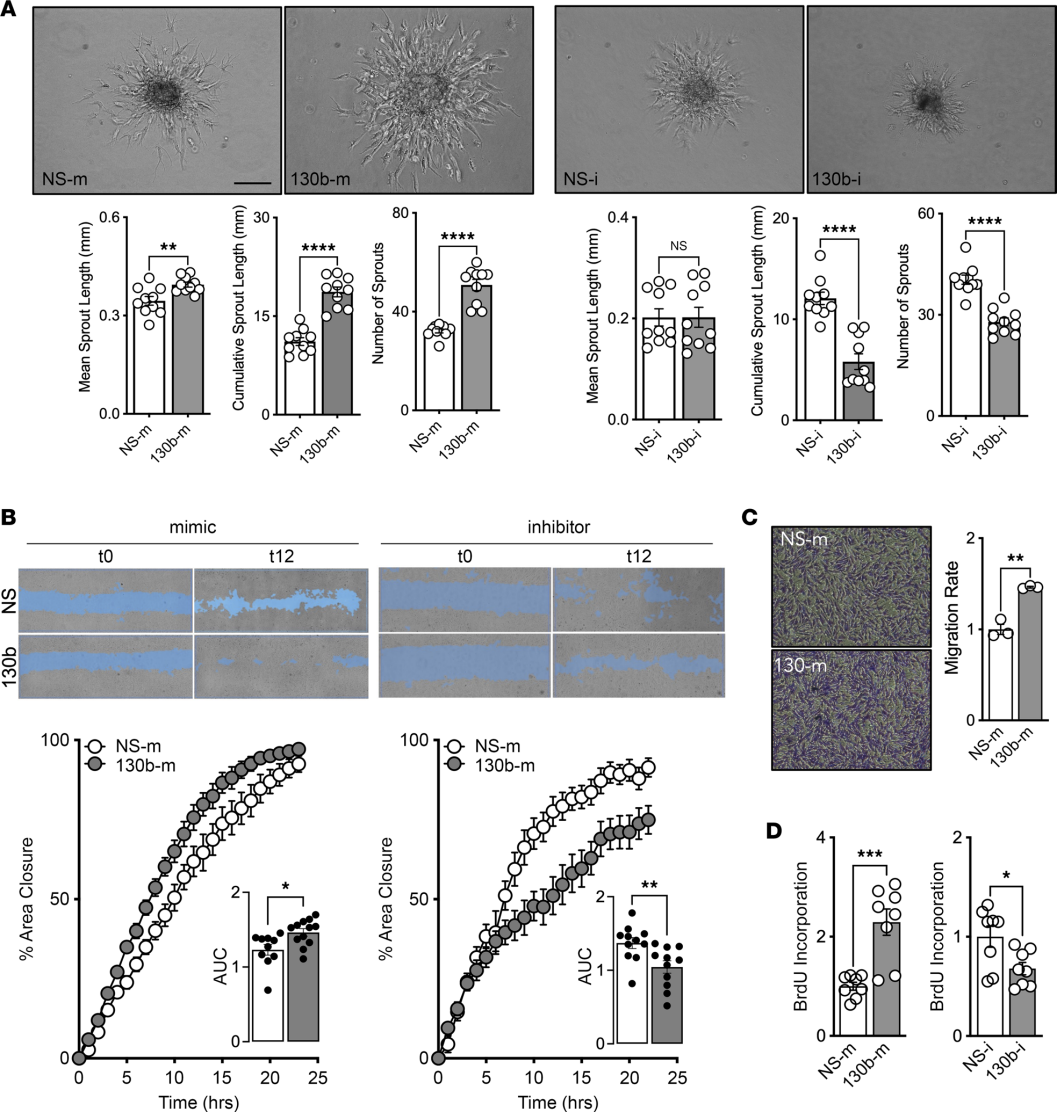
Endothelial *miR-130b* promotes angiogenesis, migration, and proliferation. HUVECs transfected with *miR-130b* or nonspecific (NS) mimics (m) or inhibitors (i) prior to cellular assays. (**A**) (Top) Representative images of 3D EC spheroid assay. (Bottom) Quantification of mean and cumulative sprout length and number of sprouts. Scale bar: 200 μm. (**B**) (Top) Representative images of scratch assay of partitioned ECs separated by a cleared area (blue). (Bottom) Images captured every hour to determine area under the curve (*n* = 12). (**C**) (Left) Representative images of ECs (purple) emerged through transwell. (Right) Normalized number of ECs in transwell migration assay (*n* = 3). Original magnification ×4. (**D**) BrdU incorporation in ECs after 5 days (*n* = 8). All statistics performed with unpaired 2-tailed Student’s *t* test. *P < 0.05, **P < 0.01, ***P < 0.001, ****P < 0.0001.

To explore the angiogenic potential of *miR-130b*, we next performed gain- and loss-of-function studies with miRNA mimetics and lock nucleic acid (LNA) inhibitors, respectively, in HUVECs. First, we performed 3-dimensional EC spheroid assays and observed that spheroids overexpressing *miR-130b* have longer and more sprouts compared with spheroids with nonspecific (NS) control mimic (Figure 2A), whereas the reverse is observed with inhibitors targeting endogenous *miR-130b*. Surprisingly, *miR-130b* inhibitors did not affect sprouting length, possibly highlighting a role in sprout formation in tip cells rather than stalk cell elongation (14). We next performed EC scratch wound assays and similarly found that ECs overexpressing *miR-130b* migrated faster, whereas inhibiting endogenous *miR-130b* slowed EC migration when compared with NS controls (Figure 2B). Overexpression of *miR-130b* also demonstrated faster migration of ECs in transwell assays (Figure 2C). Lastly, using BrdU incorporation assays, we found that *miR-130b* promoted proliferation in ECs (Figure 2D). Collectively, these cell-based assays in ECs reveal the proangiogenic potential of *miR-130b*.

### Endothelial miR-130b promotes proliferative transcriptomic networks.

**Figure 3:**
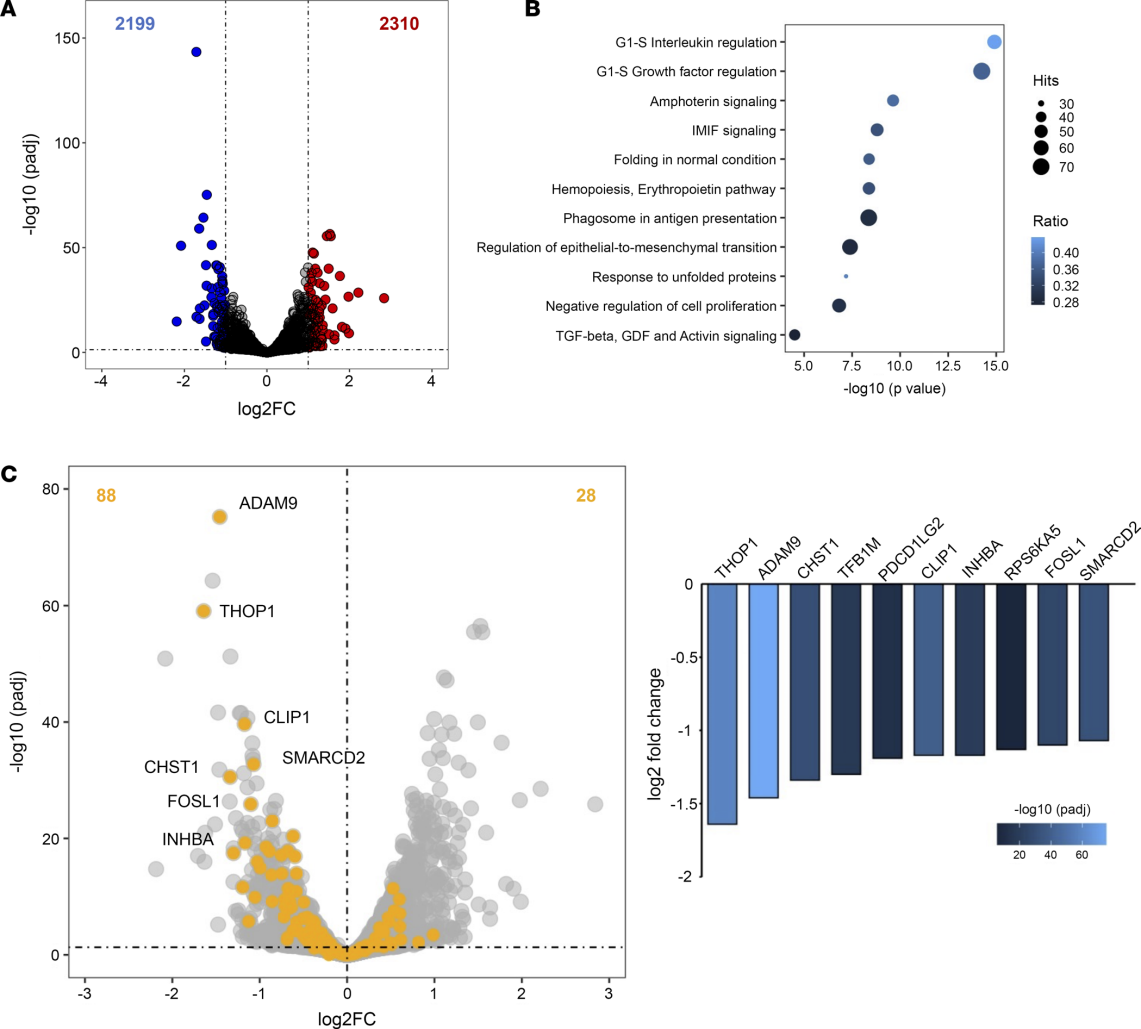
Endothelial *miR-130b* promotes proliferative transcriptomic networks. HUVECs transfected with *miR-130b* mimics compared with NS control mimics for 48 hours (*n* = 3) before RNA-Seq. (**A**) Volcano plot highlighting 2,199 downregulated and 2,310 upregulated genes in *miR-130b*–overexpressing ECs compared with NS mimic controls. (**B**) Pathway analysis of 2,310 upregulated genes organized by adjusted *P* values. (**C**) (Left) Volcano plot highlighting predicted *miR-130b* targets (yellow). (Right) List of top 10 downregulated predicted targets of *miR-130b* organized by log_2_ fold change.

To understand the potential mechanisms by which *miR-130b* promotes angiogenic activity, we performed RNA-Seq comparing HUVECs transfected with NS control or *miR-130b* mimics. Overexpression of *miR-130b* increased 2,310 genes and repressed 2,199 genes compared with controls (*P*_adj_ < 0.1) (Figure 3A). Pathway analysis of upregulated genes highlighted several cell cycling pathways including: “G1-S Interleukin regulation,” “G1-S Growth factor regulation,” and “Negative regulation of cell proliferation” (Figure 3B). This strongly supports the proangiogenic phenotype conferred by *miR-130b* overexpression in ECs (Figure 2). To identify direct targets of *miR-130b*, we integrated the dysregulated genes into 4 different predictive algorithms and identified a list of 88 downregulated genes (Figure 3C). Within the top 10 predicted *miR-130b* targets, the gene *INHBA*, a member of the TGF-β superfamily, was included; it mediates signals from either activin or inhibin protein complexes, which was of interest due to its role in inhibiting (15, 16) or promoting (17), respectively, proliferation and angiogenesis in ECs. In support, within our pathway analysis, we found “TGF-beta, GDF and Activin signaling” to be dysregulated in *miR-130b*–overexpressing ECs (Figure 3B). Furthermore, within the top predicted miR-130b targets that overlapped with the transcripts derived from RNA-Seq of *miR-130b*–overexpressing ECs, the only transcript that miR-130b mimics repressed after transient transfection studies was *INHBA* (Supplemental Figure 4).

### miR-130b targets INHBA to mediate angiogenic changes in ECs.

**Figure 4:**
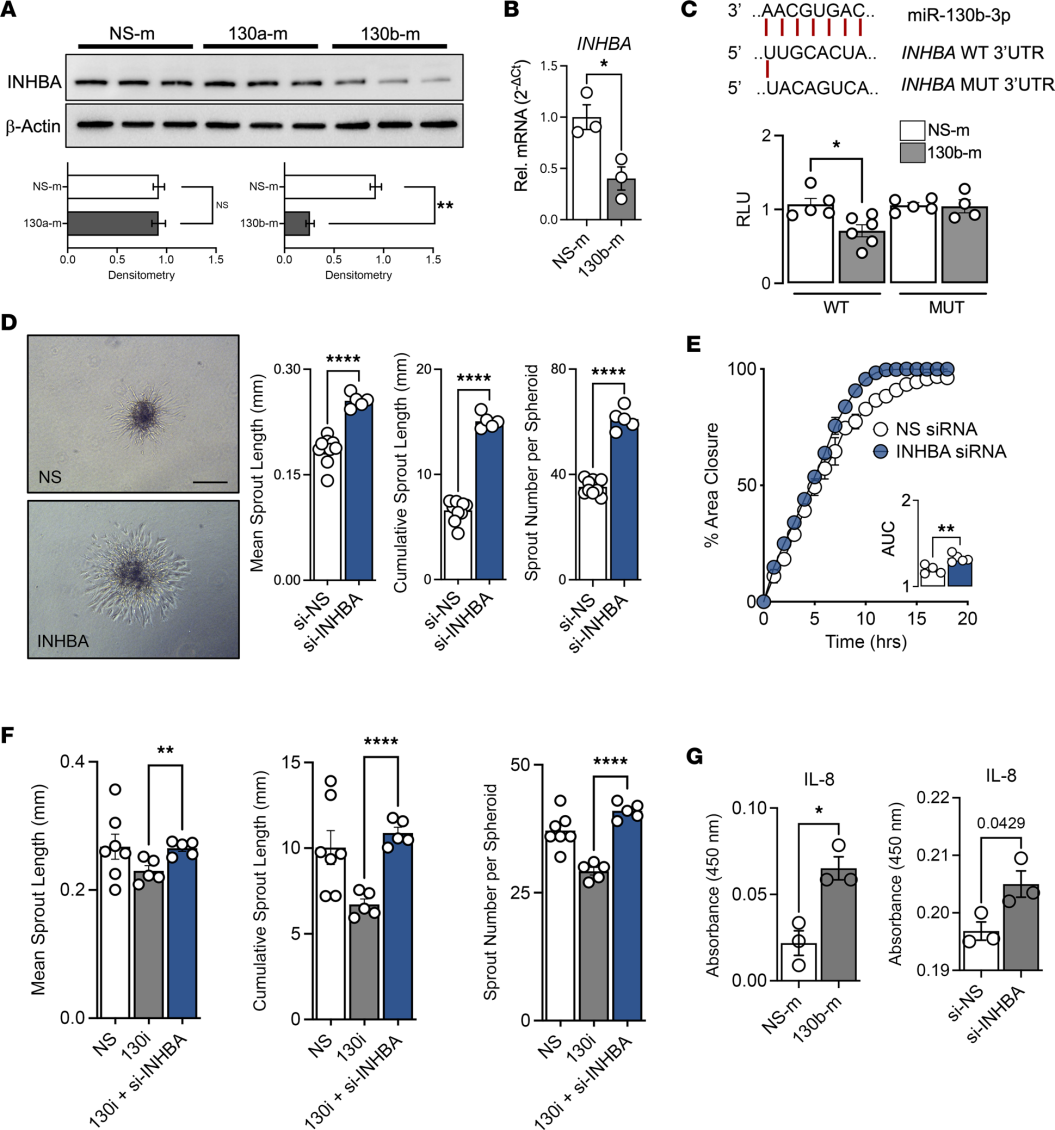
*miR-130b* targets *INHBA* to mediate angiogenic changes in ECs. (**A**) Protein abundance of INHBA in HUVECs overexpressing *miR-130a* or *miR-130b*. Densitometry normalized to β-actin (*n* = 3). (**B**) Relative expression of *INHBA* normalized to *HPRT* in HUVECs overexpressing *miR-130b* (*n* = 3). (**C**) (Top) Schematic of binding areas between *miR-130b* and *INHBA* 3′UTR. (Bottom) Relative luciferase units (RLU) of WT *INHBA* 3′UTR and MUT *INHBA* 3′UTR luciferase reporter assay with NS mimic or *miR-130b* mimic (*n* = 4–6). (**D**) (Left) Representative images of 3D EC spheroid assay. (Right) Quantification of mean and cumulative sprout length and number of sprouts. Scale bar: 200 μm. (**E**) Scratch assay of partitioned ECs captured every hour to determine area under the curve (*n* = 5). (**F**) Dependency of *miR-130b* for *INHBA*: 3D EC spheroid assay with combination of *miR-130b* inhibitors and siRNA targeting *INHBA* (*n* = 5-7). (**G**) *miR-130b* mimic or si-INHBA induce IL-8 expression by ELISA (*n* = 3). All statistics performed with unpaired 2-tailed Student’s *t* test. *P < 0.05, **P < 0.01, ****P < 0.0001.

To validate our transcriptomic analysis, we next overexpressed *miR-130b* in ECs and discovered a significant reduction of INHBA protein (Figure 4A) and transcript abundance (Figure 4B). Surprisingly, overexpression of the other miR-130/301 family members did not repress INHBA protein (Figure 4A and Supplemental Figure 5) or impact spheroid sprouting (Supplemental Figure 6). Next, we confirmed *INHBA* to be a bona fide target of miR-130b by luciferase reporter assays with the *INHBA* 3′UTR (Figure 4C). Coexpression of *miR-130b* and WT *INHBA* 3′UTR mediated repression of the luciferase reporter; however, no difference was observed with *miR-130b* and a mutant *INHBA* 3′UTR containing a scrambled 6 nucleotide change at the seed region.

To validate its putative role in EC angiogenic assays, we performed knockdown studies of *INHBA* with siRNA in ECs. Deficiency of *INHBA* in ECs promoted sprouting in 3D spheroid assays (Figure 4D) and faster closure in EC scratch wound assays (Figure 4E), which effectively phenocopied effects from *miR-130b* overexpression (Figure 2, A and B). Therefore, we next performed a combination of knockdown of *miR-130b* and *INHBA* in EC spheroids to assess for dependency. The combination of knockdown was able to rescue the decreased angiogenic potential of *miR-130b* inhibition alone (Figure 4F). To explore the potential mechanisms by which the miR-130b–mediated repression of INHBA enhances angiogenesis, we screened an angiogenesis array using protein lysates from NS-m, miR–130b-m, si-NS Ctrl, or si-INHBA transfected ECs (m, mimic; i, inhibitor; si, siRNA). Remarkably, both miR–130b-m and si-INHBA induced the expression of IL-8, a known proangiogenic chemokine, that was confirmed by ELISA (Figure 4G and Supplemental Figure 7). Collectively, *miR-130b* directly targeted *INHBA* in ECs to induce IL-8 and promote angiogenesis.

### In vivo miR-130b delivery improves revascularization in diabetic mice experiencing hindlimb ischemia.

**Figure 5:**
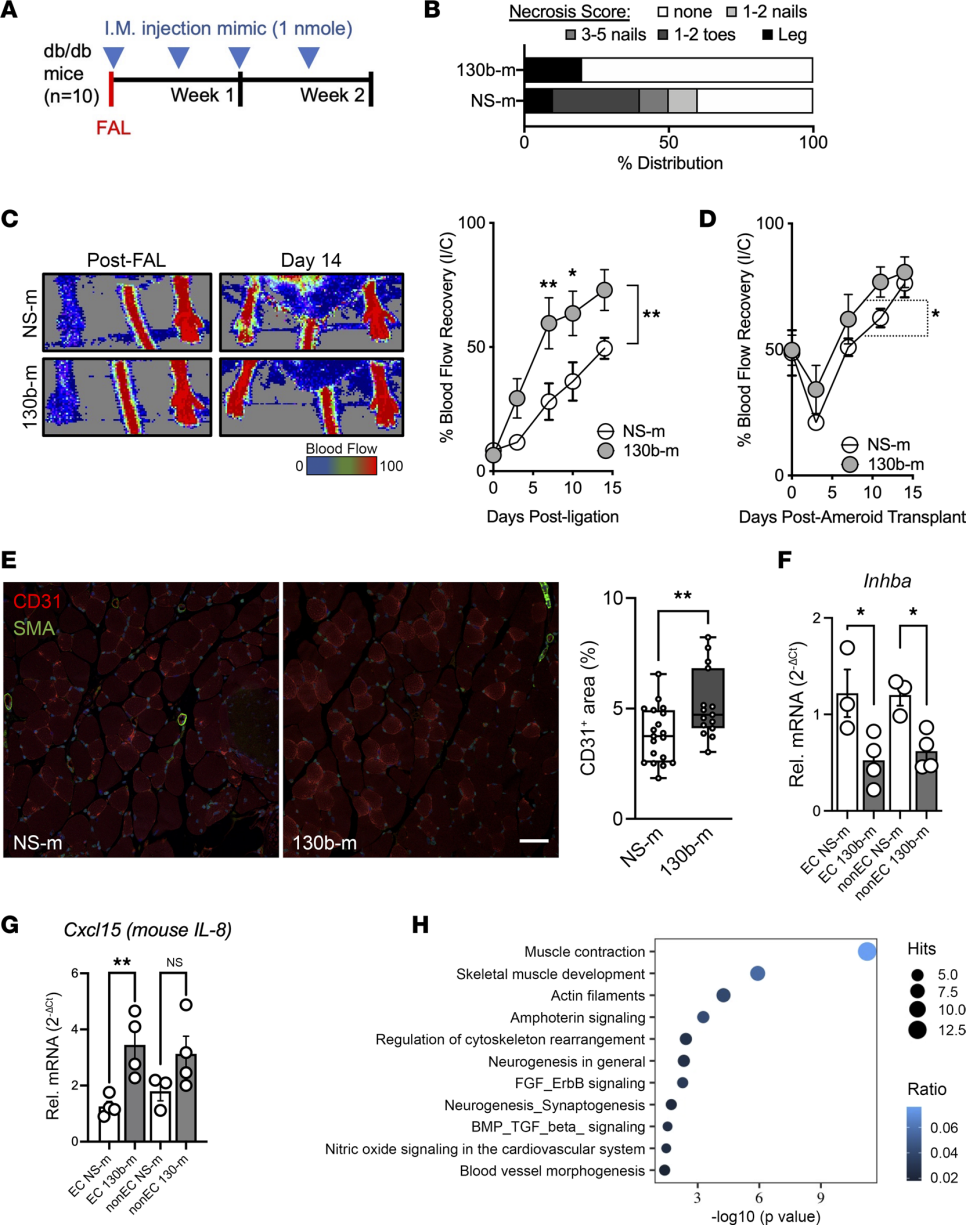
In vivo *miR-130b* delivery improves revascularization in diabetic mice experiencing hindlimb ischemia. (**A**) Schema showing experimental setup and intramuscular (I.M.) injection regimen. (**B**) Necrosis score of ischemic foot 2 weeks after FAL. (**C**) (Left) Representative LDI images of hindlimbs immediately after FAL and 14 days later. (Right) Quantification of blood flow (surgical limb/contralateral limb) by LDI images, normalized to measurement immediate after surgery, 2-way ANOVA (*n* = 8–10). (**D**) Quantification of blood flow following ameroid constrictor transplantation, 2-way ANOVA (*n* = 10). (**E**) (Left) Representative immunofluorescent images of ischemic gastrocnemius stained for SMA (green), CD31 (red), and DAPI (blue). Scale bar: 100 μm. (Right) Quantification of CD31^+^ areas per field of view (5 images per sample, *n* = 4); statistics performed with unpaired 2-tailed Student’s *t* test. (**F** and **G**) Relative expression of *Inhba* (**F**) and *Cxcl15* (**G**) normalized to *Gapdh* from CD31^+^ ECs and CD31^–^ non-ECs from ischemic gastrocnemius given NS or *miR-130b* mimics (*n* = 4). (**H**) Pathway analysis of CD31^+^ ECs compartment given NS or *miR-130b* mimics (*n* = 3). *P < 0.05, **P < 0.01.

To further evaluate the angiogenic potential of *miR-130b*, we next performed intramuscular delivery of NS control or *miR-130b* mimics into the ischemic gastrocnemius immediately following FAL surgeries in *db/db* mice and 3 more injections over the next 14 days (Figure 5A). Two weeks after FAL, we observed a reduction in necrosis scores in mice given *miR-130b* mimics compared with NS mimic controls (Figure 5B). In accordance, we also observed a robust improvement in blood flow recovery in mice given *miR-130b* mimics (Figure 5C). Interestingly, we also performed *miR-130b* mimic intramuscular delivery in *db/db* mice under subacute ischemic conditions (ameroid constrictors). While there was improvement in blood flow recovery in the *miR-130b* mimic–injected group, the differences were minor compared with the NS control group (Figure 5D). The improved blood flow recovery observed in the FAL model reflects increased angiogenesis based on elevated CD31^+^ cross-sections of the ischemic gastrocnemius muscles from *miR-130b* delivery (Figure 5E). Furthermore, *Inhba* expression was significantly reduced in ECs and non-ECs from ischemic gastrocnemius treated with *miR-130b* mimics, suggesting that the increase revascularization is a result of *Inhba* repression by ectopic *miR-130b* (Figure 5F). Interestingly, expression of IL-8 (*Cxcl15* in mice) increased in ECs and trended slightly higher in non-ECs of mice given miR-130b mimetic delivery (Figure 5G). This further supports the regulation of the proangiogenesis IL-8 by *Inhba* and *miR-130b*. Lastly, we performed RNA-Seq and pathway analysis on the EC fraction from the ischemic gastrocnemius. When comparing ECs treated with *miR-130b* mimics to NS control mimic ECs, we found 62 differentially expressed genes. Those genes highlighted several pathways, including TGF-β signaling, which supports the involvement of Inhba in the mechanism of action of *miR-130b* (Figure 5H). In summary, in vivo delivery of *miR-130b* reduced target gene *Inhba* expression and improved blood flow recovery in 2 different hindlimb ischemia models in diabetic mice.

### In vivo delivery of siRNA targeting Inhba improves revascularization in diabetic hindlimb ischemia.

**Figure 6:**
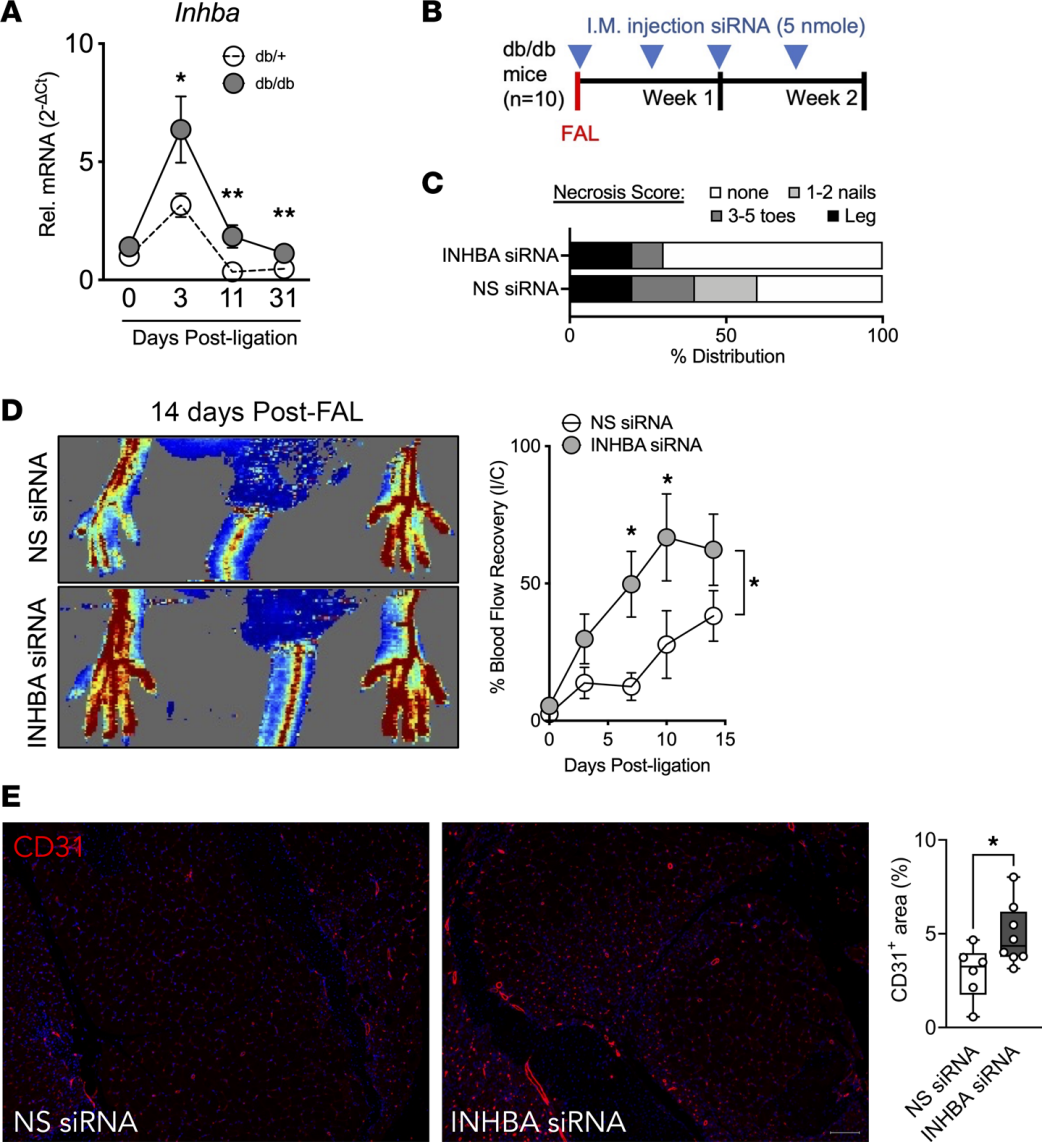
In vivo delivery of siRNA targeting *Inhba* improves revascularization in diabetic hindlimb ischemia. (**A**) *Inhba* expression normalized to *Gapdh* in ischemic gastrocnemius of *db/+* and *db/db* mice at different time points after FAL. Comparison between groups at specific time points by unpaired 2-tailed Student’s *t* test (d0, *n* = 11–12; d3, *n* = 7–12; d11, *n* = 5–6; d31, *n* = 4–7). (**B**) Schema showing experimental setup and intramuscular injection regimen. (**C**) Necrosis score of ischemic foot 2 weeks after FAL. (**D**) (Left) Representative LDI images of hindlimbs 14 days after FAL surgeries. (Right) Quantification of blood flow (surgical limb/contralateral limb) by LDI images, normalized to measurement immediate after surgery, 2-way ANOVA (*n* = 8–10). (**E**) (Left) Representative immunofluorescent images of ischemic gastrocnemius stained for CD31 (red) and DAPI (blue). Scale bar: 100 μm. (Right) Quantification of CD31^+^ areas per field of view (*n* = 6–8). Statistics performed with unpaired 2-tailed Student’s *t* test. *P < 0.05, **P < 0.01.

Expression kinetics of *Inhba* in ischemic gastrocnemius following FAL highlights 2 facets: *Inhba* was induced early (day 3), suggesting a role in inflammatory response, and induction of *Inhba* in diabetic mice was greater than in nondiabetic mice when comparing to the respective sham controls (Figure 6A). Given our data supporting a miR-130b/*Inhba*/angiogenesis axis, we performed in vivo intramuscular delivery (4 times) of *Inhba* or NS siRNAs following FAL in *db/db* mice (Figure 6B). Mice given *Inhba* siRNA experienced lower frequency of limb necrosis compared with controls 2 weeks following FAL (Figure 6C). Furthermore, knockdown of *Inhba* greatly improves blood flow to the ischemic limbs (Figure 6D); this improvement is a result of increased angiogenesis observed in the ischemic muscles (Figure 6E). Collectively, the delivery of siRNAs targeting *Inhba* phenocopies that of *miR-130b* delivery in diabetic mice experiencing hindlimb ischemia.

### miR-130b/INHBA axis observed in nondiabetic patients with PAD and CLI.

**Figure 7:**
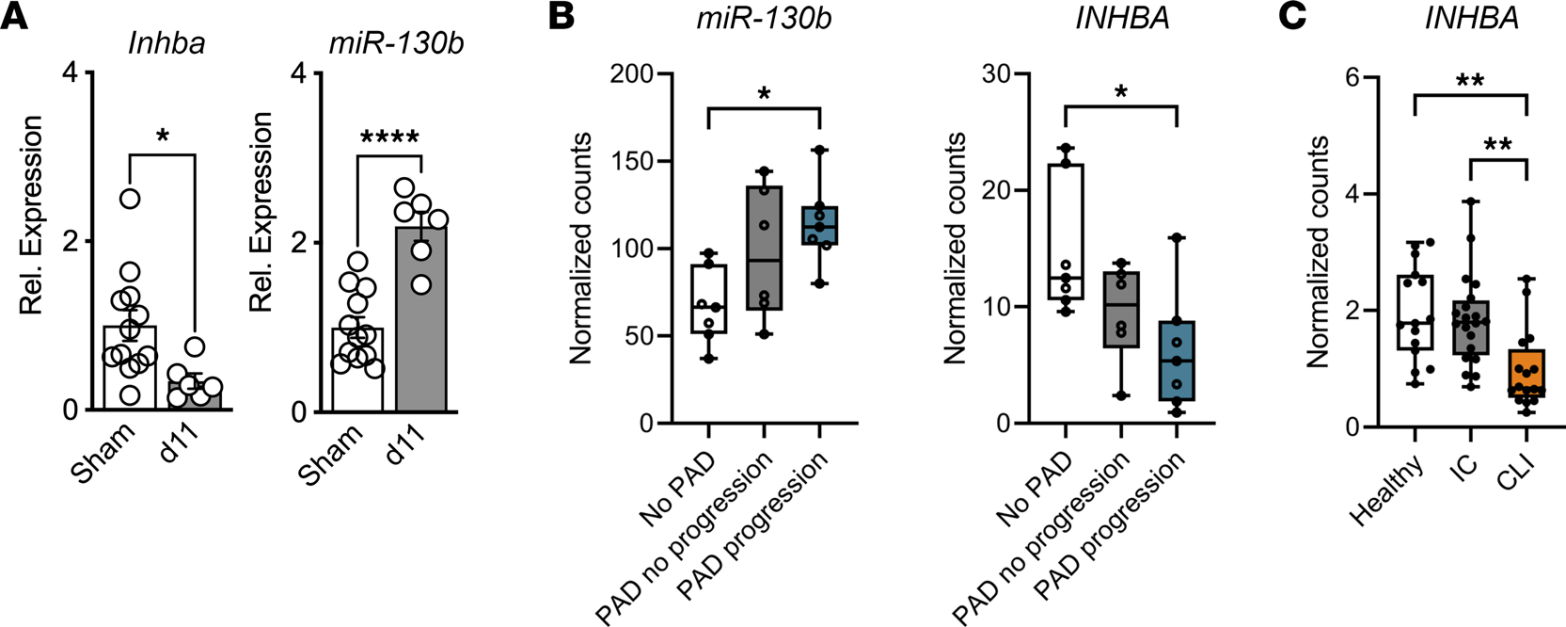
*miR-130b*/*INHBA* axis observed in nondiabetic patients with PAD and CLI. (**A**) Relative expression of *Inhba* normalized to *Gapdh* (left) and *miR-130b* normalized to *U6* (right) comparing sham control with *db/+* gastrocnemius 11 days after FAL (*n* = 6–12). (**B**) Normalized counts of *miR-130b* (left) and *INHBA* (right) from RNA-Seq of skeletal muscles from patients without PAD, with PAD, and with more severe PAD (*n* = 7). (**C**) Normalized counts of *INHBA* from RNA-Seq of skeletal muscles from patients without PAD (*n* = 15), patients with PAD with intermittent claudication (*n* = 20), and patients with CLI (*n* = 16). All statistics performed with 1-way ANOVA with multiple comparisons. *P < 0.05, **P < 0.01, ****P < 0.0001.

With our FAL animal studies, we observed an inverse relationship of *miR-130b* and *Inhba* in the gastrocnemius of nondiabetic mice (Figure 1E, Figure 6A, and Figure 7A). At day 11 following FAL, a critical time for angiogenesis process in mice, we reasoned that targeting *Inhba* by *miR-130b* is necessary to promote revascularization. This relationship is mirrored in muscle biopsies from patients with PAD as well; *miR-130b* expression increased and *INHBA* decreased with the severity of PAD (Figure 7B). The decrease of *INHBA* was also observed in the gastrocnemius muscles from an independent data set of patients in which patients with CLI had reduced *INHBA* expression but patients experiencing IC did not (18) (Figure 7C). Collectively, transcriptomic changes highlight the *miR-130b/INHBA* axis found in ischemic muscles of mice is conserved with increasing severity of PAD in patients.

## Discussion

In this study, we validated the use of plasma from patients with PAD and diabetes along with plasma from diabetic mice subjected to hindlimb ischemia models. Overlapping mouse and human data sets revealed a total of 22 commonly downregulated miRNAs, including 13 miRNAs previously characterized in hindlimb ischemia studies (6), providing affirmation of this approach to identify angiogenesis-related miRNAs. We pursued the characterization of the potentially novel *miR–130b-3p*, since it demonstrated unique expression kinetics in vivo following hindlimb ischemia in mice and in vitro under hypoxia and VEGF-A stimulation (Figure 1). In contrast, *miR-130b* appears to be suppressed by diabetic conditions, both in vivo and in vitro, thereby suggesting a common mechanism in regulating *miR-130b* expression. Furthermore, *miR-130b* overexpression can promote EC growth, even under diverse stimuli relevant to diabetic CLI, such as glucose, the free fatty acid palmitate, or hypoxia (Supplemental Figure 8). Indeed, an emerging group of angiogenic-responsive miRNAs, so-called angio-miRs, appear to be dynamically regulated by pro- or antiangiogenic stimuli in a similar manner as we and others have described, for example, for miR-26a (19), miR-126 (20), miR-135-3p (21), miR-92a (22), miR-223 (23), miR-375 (24), miR-181b (25), miR-615-5p (13), or miR-4674 (26). Interestingly, *miR-130b* expression displays an inverse relationship between plasma and ischemic tissue in patients with PAD (Figure 1, C and D). While it is a less common characteristic of miRNAs, some display similar expression patterns similar to that of *miR-130b*. For example, *miR-499* and *miR-208a* are elevated in plasma of acute myocardial infarction patients with low levels in skeletal muscles (27), and a similar inverse pattern exists for *miR-206* during skeletal muscle hypertrophy (28). While the mechanism driving this inverse relationship between circulating and tissue miRNAs is still unclear, a growing body of literature supports hypoxia-induced export of miRNAs into circulation by small extracellular vesicles, such as exosomes (29, 30). Indeed, miRNAs have recently been found to be selectively packaged into small extracellular vesicles, and this depletes the amount retained in the donor cell (31).

Development of CLI from PAD hinge on the impairment of the critical physiological processes, such as inflammation and angiogenesis. *miR-130b* has been shown to modulate oncogenic angiogenesis via targets within cancer cells (32, 33) — for example, it has modulated oncogenic angiogenesis by promoting endothelial angiogenesis by targeting *PTEN* (34). However, in the context of murine hindlimb ischemia, we did not find any significant change to *Pten* from *miR-130b* overexpression in gastrocnemius ECs or HUVEC RNA-Seq data sets (data not shown). Rather, we found *Inhba* to be the direct link of *miR-130b* to hindlimb ischemia recovery in mice. Other top-predicted targets were not affected by *miR-130b* overexpression in HUVECs, which highlights that other possible mechanisms are involved, including obstruction of seed sequence by RNA binding proteins, cell and disease context dependent stimuli, and thermodynamic properties of miRNA-mRNA hybrid interactions (35, 36). These findings further underscore the emerging recognition that miRNAs recognize target transcripts that are context specific with often vast differences in targets for the same miRNA in transformed or tumor cells compared with primary cell types (6, 9).

INHBA is a member of the TGF-β superfamily and can homodimerize to form activin A (37). The role of activin A in the context of angiogenesis had been shown to repress endothelial proliferation through Smad2/3 activation of p21 (15, 38). While INHBA was initially found in gonadal cells, multiple studies show that it is a multifunctional cytokine with potent antiangiogenic effects in various disease states from cancer, asthma, and pulmonary hypertension (16, 38, 39). Ectopic activin A, INHBA, or conditioned media containing activin A inhibited angiogenic responses of pulmonary artery ECs (16), consistent with our study demonstrating that knockdown of endogenous *Inhba* promoted a proangiogenic phenotype. While an EC-specific *Inhba*–conditional KO mouse has been generated (16), the authors did not evaluate the angiogenic potential in these mice. However, EC-specific transgenic overexpression of INHBA in mice predisposed then to the development of pulmonary hypertension without apparent differences in the lung vessel density in neonates or adults (16). Future studies will be of interest to examine these mouse models in the context of limb ischemia.

Both *miR-130b* overexpression and siRNA-mediated knockdown of *INHBA* increased IL-8 production from ECs. IL-8 is a potent proangiogenic chemokine implicated in the regulation of ischemic cardiovascular disease to tumor-associated angiogenesis (40–42). In addition to its role in leukocyte chemotaxis, angiogenic properties of IL-8 are mediated by promoting migration, proliferation, and survival in ECs (42–44). Circulating plasma levels of IL-8 were significantly higher in patients with PAD before or after exercise compared with healthy patients (45). Intramuscular injection of IL-8 increased neovascularization after hindlimb ischemia in mice by improving homing of tail vein-injected endothelial progenitor cells (46). Interestingly, Choi et al. (47) recently showed increased production of IL-8 in fibroblast growth factor 2 (FGF2)-primed human adipose–derived stem cell spheroids, which promoted angiogenesis and muscle regeneration when injected intramuscularly in a mouse hindlimb ischemia model. Collectively, these studies reinforce that a *miR-130b*/INHBA/IL-8 signaling axis may figure prominently in increasing blood vessel recovery and tissue repair after limb ischemia.

While our studies have focused on the role of the *miR130b* target *INHBA* on angiogenic paradigms in PAD/CLI, we cannot rule out a participatory role for *miR-130b* in other aspects of tissue reparative mechanisms in which *Inhba*/activin A has been implicated besides angiogenesis (15, 16, 39), such as wound healing (37) and inflammation (48). Interestingly, inflammation is dominant within the first week following FAL in mice, which is when we observed the highest expression of *Inhba* in the ischemic gastrocnemius in both *db/+* and *db/db* mice (Figure 6A). Future studies will be of interest to further explore whether *Inhba* may have a role in modulating inflammatory responses in hindlimb ischemia injury and the extent to which endothelial-specific *Inhba* expression impacts neovascularization using transgenic mouse models.

Another pathway that the *miR-130/301* family is known to regulate is the YAP/TAZ pathway (49). Indeed, we found that all members induced YAP/TAZ expression upon overexpression in ECs (Supplemental Figure 9). However, other family members did not impact angiogenic functional properties (Supplemental Figure 6), and only *miR-130b* repressed INHBA protein (Figure 4A and Supplemental Figure 5). For example, even with YAP/TAZ increased in ECs overexpressing *miR-130a*, there was no increase in sprouting with *miR-130a* overexpression (Supplemental Figure 6), suggesting that YAP/TAZ is not mediating the angiogenic effects of *miR-130b*. Future studies will be of interest to assess dependency for a range of *miR-130/301* targets using transgenic approaches.

In summary, our study identified *miR–130b-3p* from patients and mice with diabetes as a potential mediator in the development of CLI. *miR-130b* promotes rapid angiogenesis in ECs by directly targeting *INHBA*, an antiangiogenic member of the TGF-β superfamily. Ectopic local delivery of *miR-130b* or siRNA targeting *Inhba* demonstrated robust improvement in blood flow recovery and limb necrosis in diabetic mice following FAL. This demonstration of *miR-130b*/*INHBA* axis in mice and the expression correlation observed in patients with PAD highlights a therapeutic avenue to combat diabetic CLI.

## Methods

Supplemental Methods are available online with this article.

### Human samples.

Plasma for miRNA-Seq was collected from the Thrombin Receptor Antagonist in Secondary Prevention of Atherothrombotic Ischemic Events–TIMI 50 (TRA 2°P–TIMI 50) trial as described (25). Characteristics of patients are found in Supplemental Table 1.

### Animal studies.

Studies were performed in *db/+* or *db/db* mice (The Jackson Laboratory). All mice used were matched for age and sex in all experiments and maintained under SPF conditions at an American Association for the Accreditation of Laboratory Animal Care–accredited animal facility at the Brigham and Women’s Hospital. The animals were sacrificed at the end of each experimental point. If an animal appeared to be sick or suffering, it was euthanized by CO_2_ asphyxiation. These methods are consistent with recommendations from the panel on Euthanasia of the American Veterinary Medical Association.

### Statistics.

Statistical analyses were performed using GraphPad Prism version 7.0 (GraphPad Software Inc). Student’s 2-tailed *t* test was used to determine statistical significance between 2 groups. ANOVA with Bonferroni’s test was used to determine differences between more than 2 groups. Data are expressed as mean ± SEM, and *P* < 0.05 was considered significant.

### Study approval.

All protocols concerning animal use (no. 2016N000182) were approved by the IACUC at Brigham and Women’s Hospital and Harvard Medical School and conducted in accordance with the *Guide for the Care and Use of Laboratory Animals* (National Academies Press, 2011). Animal studies were performed in male db/+ or *db/db* mice (The Jackson Laboratory). All patient samples (25, 50) conform to the principles outlined in the Declaration of Helsinki and were approved by the IRB of Brigham and Women’s Hospital. All participants have given written consent to the inclusion in the study.

### Data availability.

All relevant data are available from the authors. The RNA-Seq data are accessible at GSE202856 and GSE204705.

## Author contributions

MWF and HSC conceived the hypothesis; HSC, RZ, DPC, JC, AJ, WW, and AT performed the experiments; HSC, RZ, DPC, JC, AJ, WW, TER, LS, AT, JP, PPG, MAC, MSS, MPB, and MWF designed or interpreted the results; and HSC, DPC, and MWF wrote the manuscript.

## Supplementary Material

Supplemental data

## Version 1 04/25/2023

In-Press Preview

## Version 2 05/22/2023

Electronic publication
