# Supplementary material for: Impaired angiogenesis in diabetic critical limb ischemia is mediated by a miR-130b/INHBA signaling axis
Source: JCI Insight. 2023 May 22;8(10):e163041. doi: 10.1172/jci.insight.163041 (PMC10322685; doi:10.1172/jci.insight.163041)
Supplement: Supplemental data [file jciinsight-8-163041-s041.pdf]

SUPPLEMENTAL MATERIAL

Impaired angiogenesis in diabetic critical limb ischemia is mediated by a miR-130b-INHBA signaling axis

Running Title: *Cheng et al.; miR-130b regulates diabetic critical limb ischemia*

Henry S. Cheng, PhD[1#]; Daniel Pérez-Cremades, PhD[1,2#]; Rulin Zhuang, MD[1,3#]; Anurag Jamaiyar, PhD[1#]; Winona Wu, MD[1]; Jingshu Chen, PhD[1]; Aspasia Tzani, MD, PhD[1]; Lauren Stone[4], Jorge Plutzky, MD[1]; Terence E. Ryan[4], Philip P. Goodney, MD, MS[5]; Mark A. Creager, MD[5]; Marc S. Sabatine, MD, MPH[1]; Marc P Bonaca, MD[6]; Mark W. Feinberg, MD[1*]

1 Department of Medicine, Cardiovascular Division, Brigham and Women's Hospital, Harvard Medical School, Boston, MA 02115, USA;
2 Department of Physiology, University of Valencia, and INCLIVA Biomedical Research Institute, Valencia 46010, Spain;
3 Department of Cardiothoracic Surgery, Nanjing Drum Tower Hospital, The Affiliated Hospital of Medical School of Nanjing University, Nanjing, 210029, China;
4 Department of Applied Physiology and Kinesiology, University of Florida, Gainesville, Florida, USA.
5 Heart and Vascular Center, Dartmouth-Hitchcock Medical Center and Geisel School of Medicine at Dartmouth, Lebanon, NH 03756, USA;
6 CPC Clinical Research, University of Colorado, Denver, CO 80045, USA.

#These authors contributed equally to this work.

*Corresponding author: Mark W. Feinberg, Department of Medicine, Cardiovascular Division, Brigham and Women's Hospital, Harvard Medical School, Boston, MA 02115, USA. Fax: 617-525-4380; Tel: 617-525-4381. Email: mfeinberg@bwh.harvard.edu

Methods

Human samples

Plasma for miRNA-sequencing was collected from the Thrombin Receptor Antagonist in Secondary Prevention of Atherothrombotic Ischemic Events (TRA 2°P)-TIMI 50 trial. Characteristics of human subjects are found in **Supplemental Table 1**.

Skeletal muscle biopsies were obtained from the gastrocnemius muscle of CLI patients within the confines of the operating room via a percutaneous muscle biopsy using sterile procedures. A portion of the muscle was quickly trimmed of fat/connective tissue and frozen in liquid nitrogen cooled isopentane for histochemical analysis. All study procedures were carried out according to the Declaration of Helsinki and participants were fully informed about the research and informed consent was obtained and procedures were approved by the institutional review boards at the University of Florida.

RNA In Situ Hybridization

hsa-miR-130b-3p probe labeled with FAM (5' and 3') was ordered from Qiagen (GeneGlobe ID – YD00615198). Detection of endogenous hsa-miR-130b-3p in frozen human gastrocnemius biopsy sections was carried out as described by Nielsen et al[1] with some modifications. A Rabbit anti-Fluorescein/Oregon Green polyclonal antibody (catalog # A-889, ThermoFisher) was used to detect the FAM-labeled probe, followed by signal amplification using the AlexaFluor 488 Tyramide SuperBoost kit (catalog #B40922, ThermoFisher). Sections were co-stained with AlexaFluor 647 anti-CD31 antibody (catalog #ab215912, Abcam) at a 1:200 dilution overnight at 4C. ProLong Gold antifade mountant with DAPI (catalog #P36935, ThermoFisher) was applied and slides were coverslipped.

Animal studies

Studies were performed in *db/+* and *db/db* mice (The Jackson Laboratory). All mice used were age-matched and sex-matched in all experiments and maintained under SPF conditions at an American Association for the Accreditation of Laboratory Animal Care-accredited animal facility at the Brigham and Women's Hospital. Animal protocol

(#2016N000182) were approved by the Institutional Animal Care and Use Committee at Harvard Medical School and conducted in accordance with the National Institutes of Health Guide for Care and Use of Laboratory Animals.

Hindlimb ischemia mouse models

Mice were subjected to two different surgeries to replicate critical limb ischemia: 1) femoral artery ligation, which causes immediate cessation of blood flow hence (Acute ischemia); and 2) ameroid constrictors which gradually expands from fluid adsorption inducing artery occlusion (Sub-acute ischemia). Briefly, mice were injected i.p. with 150 μl of 20% ketamine/5% xylazine in 0.9% saline. Once anesthetized, the right medial thigh to the suprapubic area was treated with a commercial emollient to remove fur and sterilized with Povidone iodine. Skin and fascia were dissected away to the femoral bed. In the acute hindlimb ischemia model, femoral artery and surrounding tissue was proximally and distally ligated with 7-0 Prolene sutures. The arterial bed in between sutures was cauterized. Abrogation of blood flow compared to the contralateral limb (<10%) was confirmed using a laser Doppler imager (Moor Instruments, UK). Sub-acute hindlimb ischemia model was performed using ameroid constrictors, which induce gradual femoral artery occlusion over 1-3 days. Two ameroid constrictors were placed on the femoral artery, one proximal to the lateral circumflex femoral artery and the second proximal to the bifurcation of the popliteal and saphenous arteries. Both constrictors were positioned with the slot facing up, ensuring proper setting of the artery within the constrictor. Mice were sutured closed at the level of the fascia and subsequently, the skin. Sham-treated mice were treated the same way except once the femoral artery was visualized, the incision was closed without ligation of the femoral artery or ameroid constrictor addition. Percent blood flow recovery was calculated by comparing a ratio of ischemic paw to contralateral paw Doppler count profiles and normalized blood flow recovery was calculated by comparing the ratio of ischemic to contralateral paw Doppler count profiles to day 0 post-operative percent blood flow.

Endothelial Cell Isolation

Gastrocnemius muscles were grinded with scissors and digested by using 1 mg/ml Collagenase type 2 (Worthington Biochemical LS004177) and 1 mg/ml Dispase II (Roche, 04942078001) and incubated at 37°C for 40 minutes. Digestion was neutralized with DMEM/F12 medium containing 10% FBS, followed by centrifugation at 500g for 10 min at 4℃. The slurry was passed through cell strainers (Corning Falcon/Westnet). After centrifugation, the cell pellet was re-suspended in incubation buffer (PBS pH 7.2, 0.1% BSA, 2mM EDTA, 0.5% FBS). Endothelial cells were captured using magnetic Dynabeads (sheep antirat IgG, Invitrogen, 00412289) conjugated with rat anti-mouse CD31 antibody (BD Biosciences, 557355) at a ratio of 5:1 Dynabeads/antibody and allowed to tumble at 4°C for 20 minutes. The slurry of lysate and Dynabead/antibody mixture was bound on a Dynamag-2 Magnet (Invitrogen) for 1 minute and the supernatant was collected as a non-endothelial cell fraction. The beads containing bound endothelial cells were then washed on the Dynamag-2 Magnet five times using wash buffer (PBS pH 7.2, 0.1% BSA) and the resultant pellet was collected as an endothelial cell fraction.

Plasma miRNA sequencing

The EdgeSeq miRNA Whole Transcriptome Assay from HTG Molecular Diagnostics, Inc. (AZ, USA) was used to measure miRNA expression in plasma from human donors and mice. The HTG EdgeSeq system combines quantitative nuclease protection assay chemistry with a next-generation sequencing platform to enable the semi-quantitative analysis of 2,083 human miRNA transcripts in a single assay. Fifteen microliters of plasma were used for extraction-free sample processing and quantitative nuclease protection assay using the EdgeSeq processor (HTG Molecular Diagnostics, Inc.). The libraries were sequenced using Illumina NextSeq, and data were parsed through HTG EdgeSeq before count data were assessed for quality and analyzed using R.

Bulk RNA-Seq analysis and pathway enrichment analysis.

RNA library preparation and sequencing analysis was conducted at GENEWIZ, LLC./Azenta US, Inc (South Plainfield, NJ, USA). The RNA sequencing libraries were

prepared using the NEBNext Ultra II RNA Library Prep Kit for Illumina using manufacturer's instructions (New England Biolabs, Ipswich, MA, USA). The sequencing libraries were multiplexed and clustered onto a flowcell and loaded onto the Illumina HiSeq instrument according to manufacturer's instructions. The samples were sequenced using a 2x150bp Paired End configuration. Trimmed reads were mapped to the reference genome available on ENSEMBL using the STAR aligner v.2.5.2b. The mean quality score of all samples was 38.61 with a range of 42,000,000-68,000,000 reads per sample. All samples had > 92% of mapped fragments over total fragments. Unique gene hit counts were calculated by using feature Counts from the Subread package v.1.5.2. Total gene hit counts and CPM values were calculated for each gene and downstream differential expression analysis between specified groups was performed using DESeq2. Genes with adjusted p-value < 0.05 were labeled as differentially expressed genes for each comparison. Differentially expressed genes were subjected to gene set enrichment analyses by using MetaCoreTM (Clarivate) software. Enrichment analysis for functional ontologies (Process Networks) and analysis using network building tools was performed in MetaCoreTM. A false discovery rate (FDR) < 0.05 was used as threshold for significance in enrichment analysis. Visualization of pathway enrichment analysis were performed as dotplot using (ggplot2 package).

miRNA target prediction

Differentially expressed genes (DEGs) were identified as being at least 2.0-fold change and adjusted p-value < 0.05. DEGs were subjected to target identification by using Ingenuity Pathway Analysis (IPA winter release Dec 2020, Qiagen) software. IPA microRNA Target Filter tool was used for miRNA target prediction. Different miRNA target prediction programs (TargetScan, miRecords, Ingenuity Knowledge Base and TarBase) filtered our miRNA-mRNA pairings. Confidence filter was used by selecting both experimentally observed and predicted target correlations.

Cell Culture and Transfection

Human umbilical vein endothelial cells (HUVECs; Lonza) were cultured in endothelial cell growth medium EGM-2 (Lonza, CC-3162). Cells that were utilized for experiments were

passaged no more than six times. bEnd.3 cells (ATCC, CRL-2299) were cultured in Dulbecco's Modified Eagle Medium/F12(1:1) (DMEM; Gibco, 11320-033) supplemented with 10% fetal bovine serum (FBS) and 1% Penicillin-streptomycin (P/S). HEK293T cells (ATCC, CRL-3216) were cultured in Dulbecco's Modified Eagle Medium (DMEM) supplemented with 10% FBS and 1% P/S. For culture bone marrow-derived macrophages (BMDMs), bone marrow was isolated from the femur mice and cultured in Iscove's Modified Dulbecco's Medium (IMDM; Sigma, I3390) supplemented with 20 ng/ml recombinant mouse M-CSF (macrophage colony stimulation factor) (R&D Systems, 416-ML-10), 10% FBS and 1% P/S. Medium was changed every 3 days and cells were used after 7 days in culture. Transfection was performed using Lipofectamine 2000 (Invitrogen) as described in the manufacturer's protocol. Negative control inhibitor (4464076), hsa-miR-130b-3p inhibitor (4464084, MH10777), negative control mimic (4464058), hsa-miR-130b-3p mimic (4464066, MC10777), negative control siRNA (4390843), and INHBA siRNA (4390771, s68336) are all from Thermofisher Scientific and used for transfection at 50 nM in HUVECs.

RNA Isolation and real-time quantitative PCR

Total RNA was extracted by using Trizol reagent following the manufacturer's protocol (Invitrogen, 15596-026). The concentration and quality control of RNA was examined using NanoDrop 2000 (ThermoFisher). miRNAs were reverse transcribed using either miScript reverse transcription kit from Qiagen (218061) and miRCURY LNA miRNA PCR assay (339306) according to the manufacturer's instructions, or High-Capacity cDNA Reverse Transcription Kit (Thermofisher, 4368814; TaqMan, 130a #000454; 130b #000456, 301a #000528, 301b #002392). miScript SYBR Green PCR Kit (Qiagen, 218073) or Taqman Universal Master Mix II, with UNG (Thermofisher 4440042) was used for quantitative real-time PCR analysis with the AriaMx real-time PCR system (Agilent Technologies) or Quantsudio 6 Pro (Thermofisher) following the manufacturer's instructions. *miR-130a-3p (*#000454) *miR-130b-3p* (YP00204317; #000456), *miR-301a-3p (*#000528), and *miR-301b-3p (*#002392) expression levels were normalized to *U6* snRNA (YP02119464; #001973) and were calculated using $2^{-\Delta Ct}$ method. cDNA for mRNA

was produced using High-Capacity cDNA Reverse Transcription Kit (Thermofisher, 4368814). mRNAs expression levels were normalized to *HPRT* or *Gapdh* and were calculated using $2^{-\Delta Ct}$ method. Subsequent RT-qPCR was performed using GoTaq qPCR Master Mix (Promega). List of primers in **Supplemental table 2**.

Immunofluorescence staining

For immunofluorescence staining, cells were fixed in 4% PFA (Boston Bio Products) for 24h and embedded in paraffin for sectioning. Slide sections were blocked with 5% donkey serum (Jackson ImmunoResearch Lab) for 1 h and then incubated with primary antibodies, CD31 (1:50, Dianova, DIA310) and αSMA (1:500, Sigma-Aldrich, A5228) overnight at 4 ºC. Slides were washed and incubated with conjugated secondary antibodies (Jackson ImmunoResearch Lab) Cy3 conjugated donkey anti-rat secondary antibody (1:300, Cat#: 712-165-153) and Alexa 647 conjugated donkey anti-rabbit secondary antibody (1:300, Cat#: 711-605-152) and Alexa 488 conjugated donkey anti-rabbit secondary antibody (1:300, Cat#: 711-545-152) for 90 min at room temperature. Cell nuclei were stained with 4′,6-diamidino-2-phenylindole (DAPI). Immunofluorescence imaging was performed by BIDMC confocal imaging and IHC core facility. Images were acquired on a Carl Zeiss LSM 880 confocal microscope using Zen black software version 2.3 SP1 (BIDMC confocal imaging and IHC core facility). Objective lenses 10x 0.45 NA and 20x 0.8 NA were used for image acquisition.

Western blot

Cells were lysed in RIPA buffer (ThermoFisher Scientific, USA) containing 1% protease and phosphatase inhibitors and resolved by SDS-PAGE. The proteins were separated by gel electrophoresis and then transferred onto PVDF membranes (Bio-Rad, USA). The membranes were blocked with 5% non-fat milk in 1X TBST at room temperature for 1 h and incubated overnight at 4 °C with antibodies against INHBA (AB128958, 1:1000; Abcam), GAPDH (D16H11, 1:1000; Cell Signaling), Beta-Actin (8H1-D10, 1:2000; Cell Signaling), phospho-SMAD2 Ser456/467 (138D4, 1:1000; Cell Signaling), SMAD2

(D43B4, 1:1000; Cell Signaling), Membranes were incubated with secondary antibody for 1 h at room temperature. Protein bands were detected by enzyme-linked chemiluminescence using a luminescent image analyzer (Bio-Rad, Chemidoc).

Angiogenesis Protein Array

Cells were lysed in RIPA buffer (ThermoFisher Scientific, USA) containing 1% protease and phosphatase inhibitors. Protein lysates were used on RayBio C-series human angiogenesis antibody array 1 kit (AAH-ANG-1-2) as per manufacturer's instructions. Protein dots were detected by enzyme-linked chemiluminescence using a luminescent image analyzer (Bio-Rad, Chemidoc).

In vitro functional assays

Scratch assays were performed seeding 20,000 HUVECs per well in 35 mm µ-dish glass plates (Ibidi, 501149017) in EGM2 media containing growth factors and 50 ng/µl VEGF (R&D Systems, 293-VE/CF). When cells were confluent, well-dividers were removed and time 0 image was obtained. Imaging was taken every hour up to 24 hours using CytoSMART Omni (Cytosmart). For EC spheroid sprouting assay, HUVECs were cultured overnight in hanging drops on nonadherent plastic dishes in EBM-2 medium with 0.2% methylcellulose (Sigma-Aldrich) using 1,000 cells/spheroid. Spheroids were embedded in a collagen matrix and incubated for 24 hours with culture media on top (50:50) and supplemented with VEGF (50 ng/µl). In some experiments, cells were treated with recombinant activin A (R&D Systems, 338-AC-010/CF) at 100 ng/mL for 24 hours. Number of sprouts and total sprout length of 5-10 spheroids per condition were used for data analysis by using NIH ImageJ software.

BrdU Proliferation Assay

For assessment of cell proliferation with high glucose, proliferation assays were performed by culturing HUVECs or murine skeletal muscle ECs in EGM-2 containing 25mM of mannitol or D-glucose for 24 hours, and then seeding 4,000 cells per well in 96-well plates. 16 hours later, cells were labeled with BrdU labeling reagent and placed in either in normoxic conditions (21%

O2) or hypoxic conditions (2-3% O2) for 8 hours. For assessment of cell proliferation with palmitate (Sigma-Aldrich, P0500-10G), proliferation assays were performed by culturing HUVECs or murine skeletal muscle ECs in EGM-2 media, and then seeding 4,000 cells per well in 96-well plates in EGM-2 containing 10mM of BSA or palmitic acid. After 16 hours, cells were labeled with BrdU labeling reagent and placed in either in normoxic conditions (21% O2) or hypoxic conditions (2-3% O2) for 8 hours. Cells were subsequently fixed and quantitated using the Cell Proliferation ELISA BrdU Colorimetric Kit according to the manufacturer's instructions (Roche, 11647229001).

Luciferase Reporter Assay

Inhba 3'UTR reporter generated from Genecopoeia (MmiT093015-MT05; NM_008380.2). HEK293T cells co-transfected with 2μg luciferase plasmid vector with 50 nM *miR-130b* mimic or negative control mimic for 24 hours. Analysis of luciferase activity with Dual-Luciferase Reporter Assay System (Promega, E1910) and standard 96-well plate reader.

Statistics

Statistical analyses were performed using GraphPad Prism version 7.0 (GraphPad Software Inc). Student t test was used to determine statistical significance between two groups. ANOVA with Bonferroni's test was used to determine differences between more than two groups. Data are expressed as mean ± SEM, and results were considered as significantly different using $P<0.05$.

Data availability

All relevant data are available from the authors. The RNA-seq data are accessible at: GSE202856 and GSE204705. Source data are provided with this paper.

Reference:

[1]Nielsen, B.S., Møller, T., Holmstrøm, K. (2014). Chromogen Detection of microRNA in Frozen Clinical Tissue Samples Using LNA™ Probe Technology. In: Nielsen, B. (eds) In

Situ Hybridization Protocols. Methods in Molecular Biology, vol 1211. Humana Press, New York, NY. https://doi.org/10.1007/978-1-4939-1459-3_7

Supplemental Figures

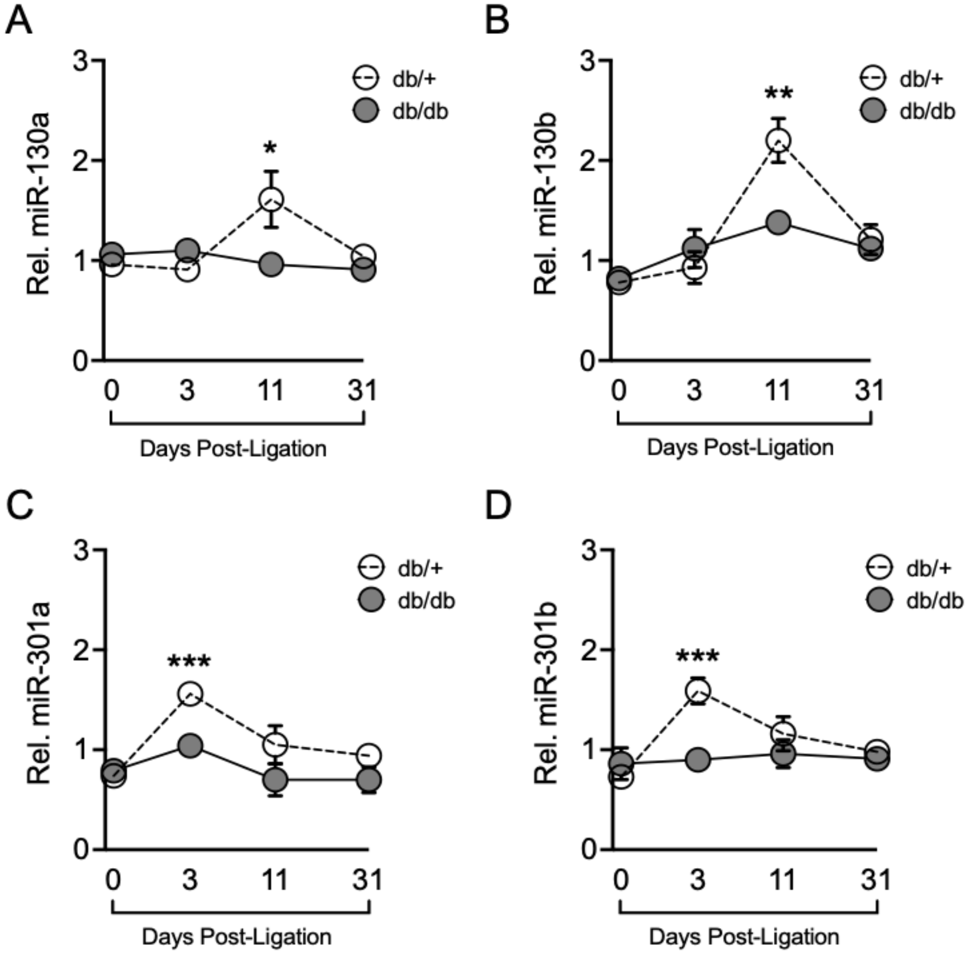

Supplementary Figure 1. Expression of *miR-130/301* family members in experimental PAD in db/+ and db/db mice.

Expression of (A) *miR-130a*, (B) *miR-130b*, (C) *miR-301a*, and (D) *miR-301b* normalized to *U6* in ischemic gastrocnemius of *db/+* and *db/db* mice at different time points post-FAL. Comparison between groups at specific time points by unpaired 2-tailed student t-test. *$p < 0.05$, **$p < 0.01$, ***$p < 0.001$.

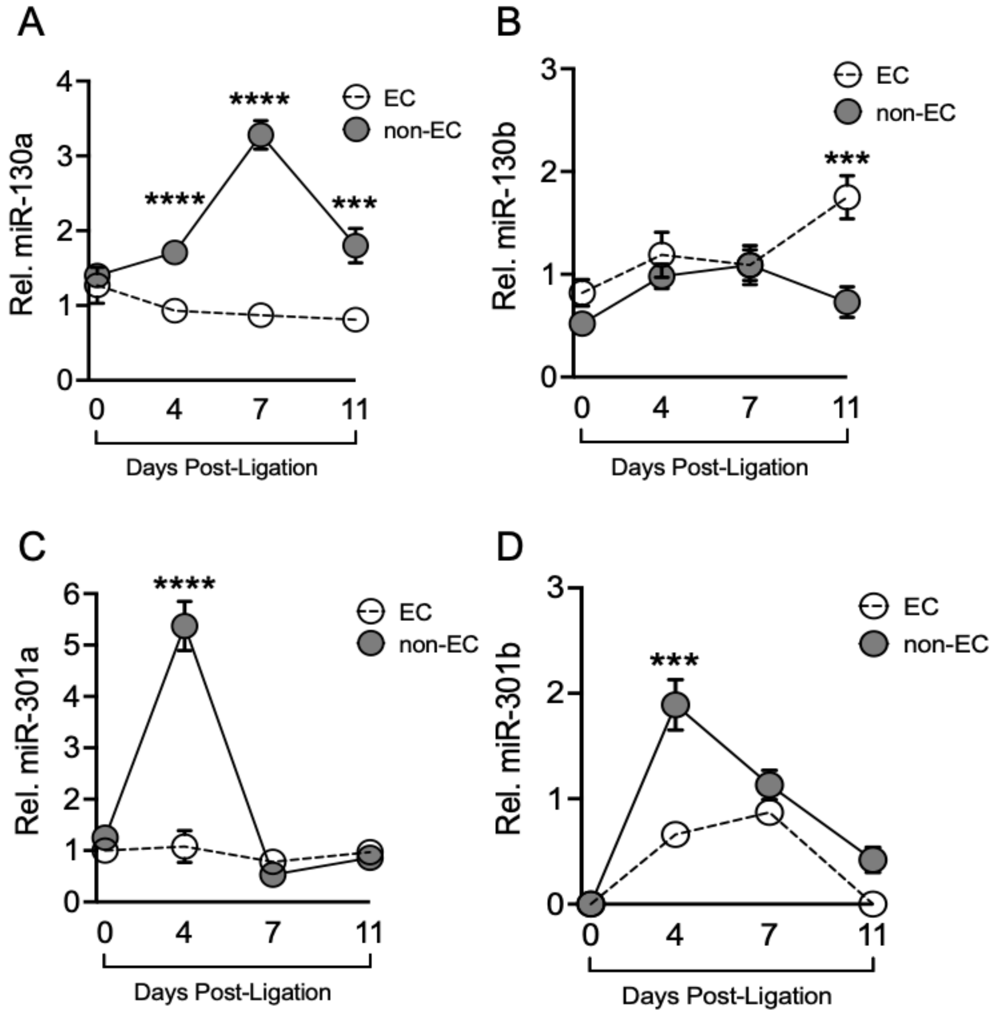

Supplementary Figure 2. Expression of *miR-130/301* family members in EC and non-EC fractions in murine experimental PAD model.

Expression of (A) *miR-130a*, (B) *miR-130b*, (C) *miR-301a*, and (D) *miR-301b* normalized to *U6* in EC and non-EC fractions of gastrocnemius in *db/+* mice at different time points post-FAL. Comparison between groups at specific time points by unpaired 2-tailed student t-test. ***$p < 0.001$, ****$p < 0.0001$.

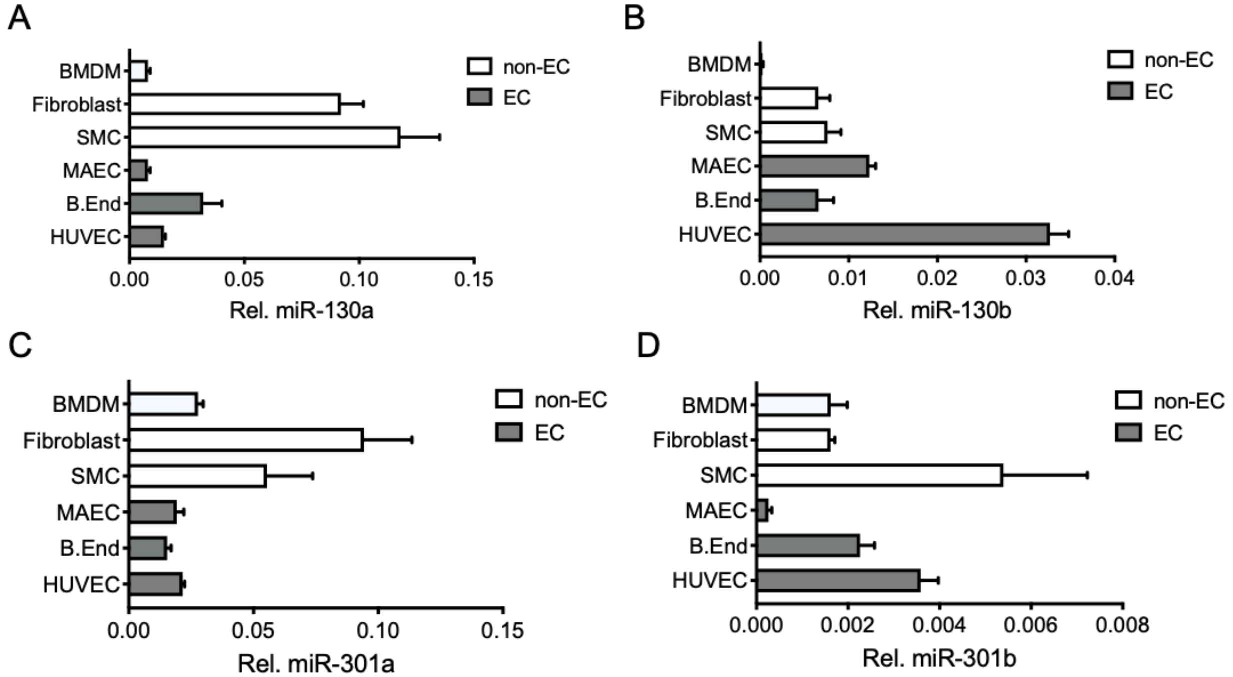

Supplementary Figure 3. Cellular expression of miR-130/301 family members. Expression of **(A)** *miR-130a*, **(B)** *miR-130b*, **(C)** *miR-301a*, and **(D)** *miR-301b* normalized to *U6* in a variety of mouse (Fibroblast; BMDM macrophages; VSMC vascular smooth muscle cell; MAEC aortic EC; B.End3 brain EC) and human (HUVEC umbilical vein EC) cell lines.

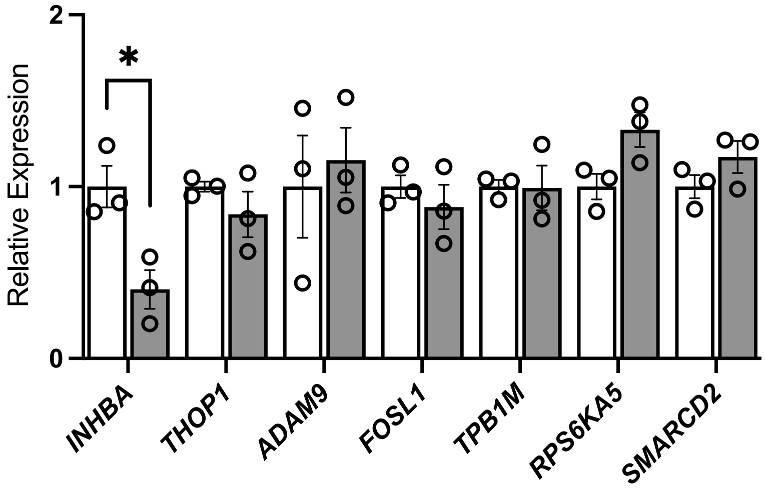

Supplementary Figure 4. Expression of predicted targets of *miR-130b.*

Expression of top downregulated predicted targets of *miR-130b*. HUVECs transfected with negative control mimic (white bar) or *miR-130b* mimic (gray bars). Unpaired 2-tailed student t test was performed (n=3), *p<0.05.

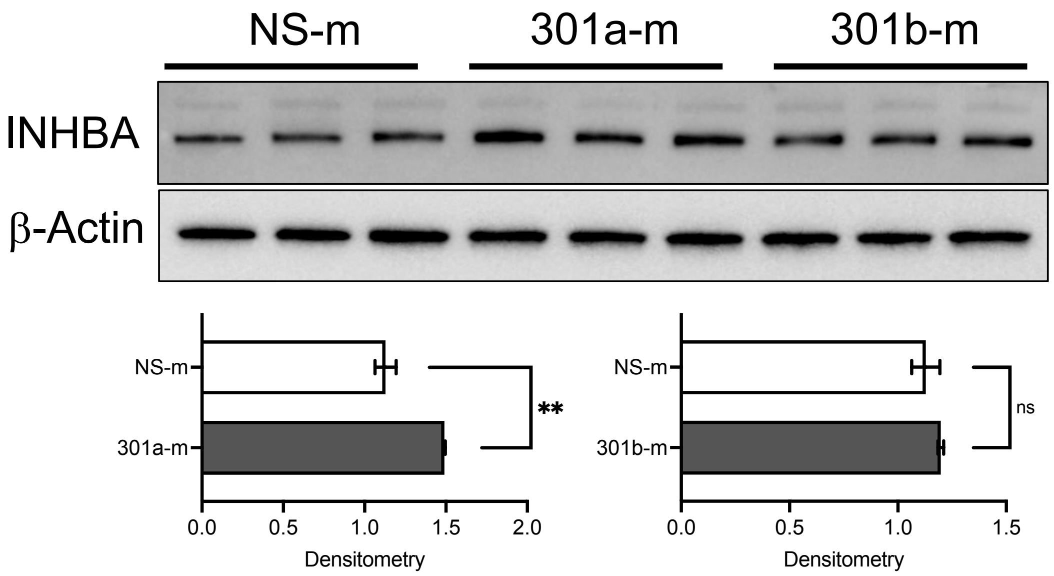

Supplementary Figure 5. *miR-301a* and *miR-301b* does not repress INHBA in ECs

Protein abundance of INHBA in HUVECs overexpressing *miR-301a* or *miR-301b*. Densitometry normalized to β-Actin (n=3). Comparison to non-specific (NS) mimic group by unpaired 2-tailed student t test was performed (n=3), **p<0.01.

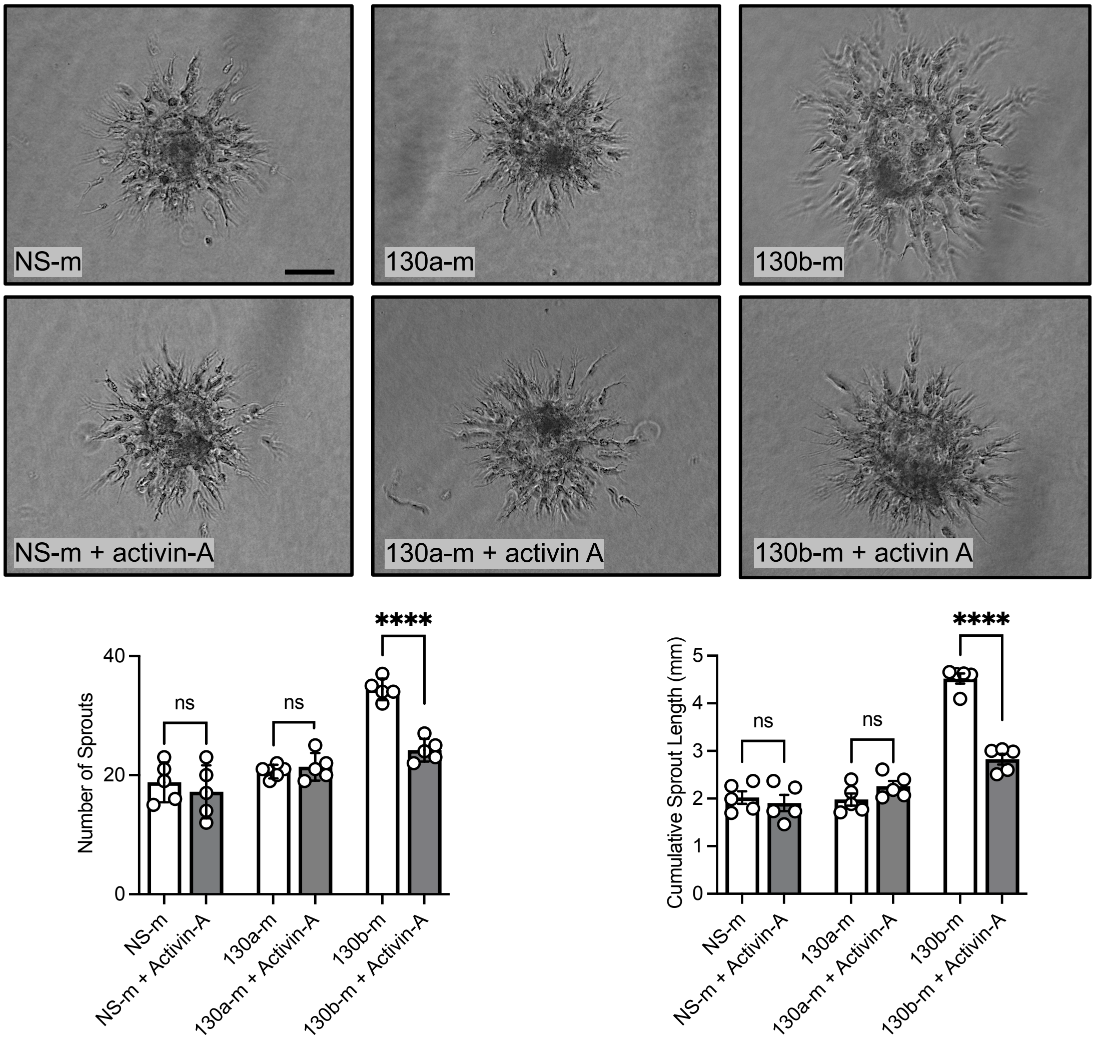

Supplementary Figure 6. Overexpression of *miR-130a* was ineffective at regulating angiogenesis. HUVECs transfected with non-specific (NS), *miR-130a*, or *miR-130b* mimics (m) were treated with or without 100 ng/mL Activin A and spheroid formation was quantified (gray bars). (Top) Representative EC spheroid images with 200 μm scale bar. (Bottom) Quantification of sprout number and cumulative sprout length. Comparison between groups by unpaired 2-tailed student t-test (n=5). ****$p < 0.0001$.

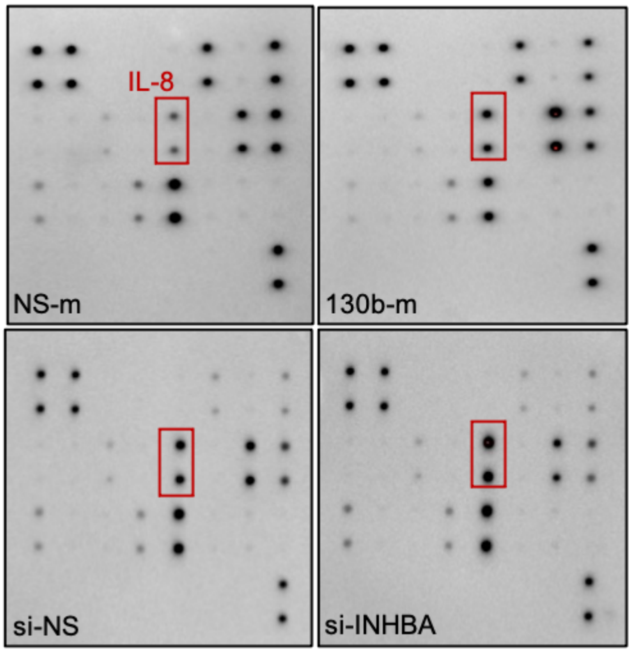

Supplementary Figure 7. *miR-130b* overexpression or si-*INHBA* increase expression of IL-8.
Protein lysates from NS-m, *miR-130b*-m, si-NS Ctrl, or si-*INHBA* transfected ECs were used on RayBio C-series human angiogenesis Ab array. Red boxes indicate IL-8 technical duplicates.

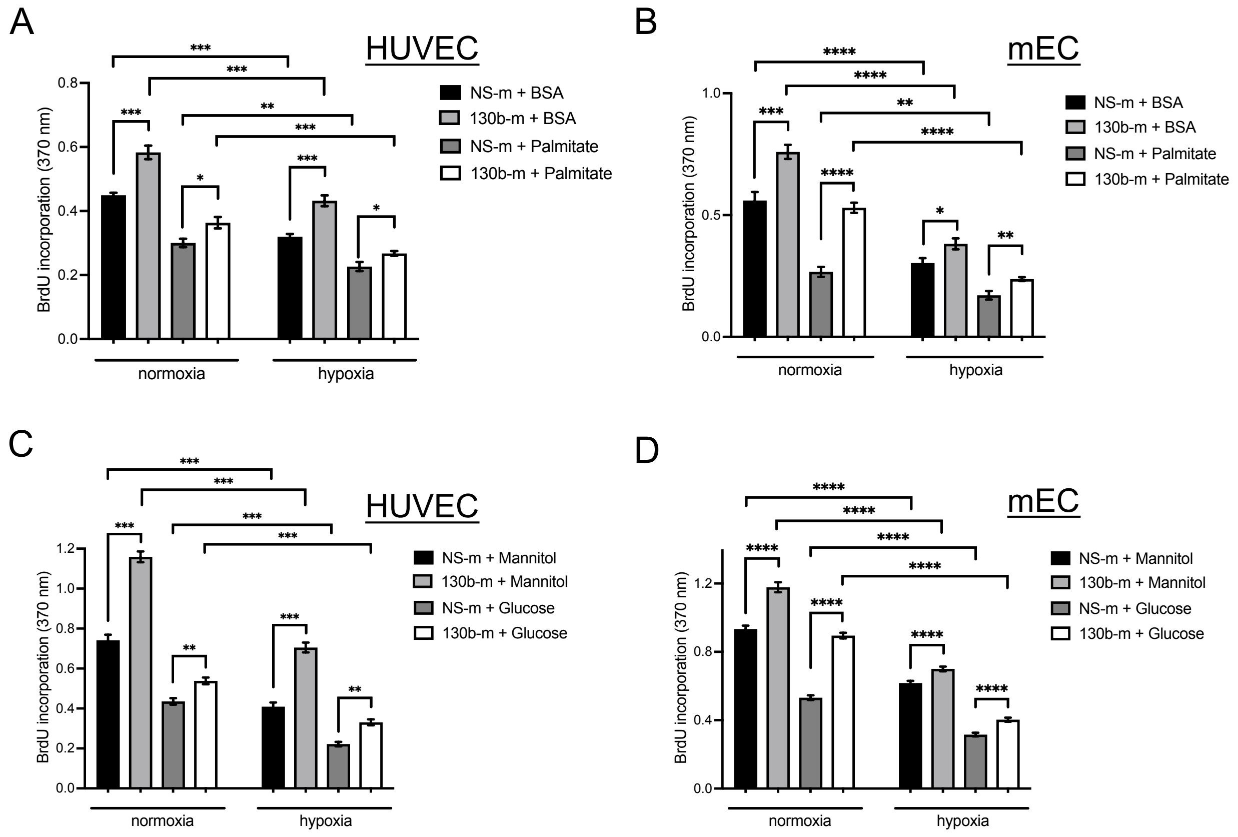

Supplementary Figure 8. Overexpression of <u>*miR-130b*</u> promotes endothelial cell growth in response to glucose, the free fatty acid palmitate, or hypoxia. Overexpression of *miR-130b* in HUVEC (**A** and **C**) and murine skeletal muscle ECs (mECs) (**B** and **D**) under normoxic and hypoxic conditions. **(A-B)** ECs treated with palmitate for 24h with BSA as control (n=6). **(C-D)** ECs treated with glucose for 48h with mannitol as control (n=6). Comparison between groups by unpaired 2-tailed student t-test. *p < 0.05, **p < 0.01, ***p < 0.001, ****p < 0.0001.

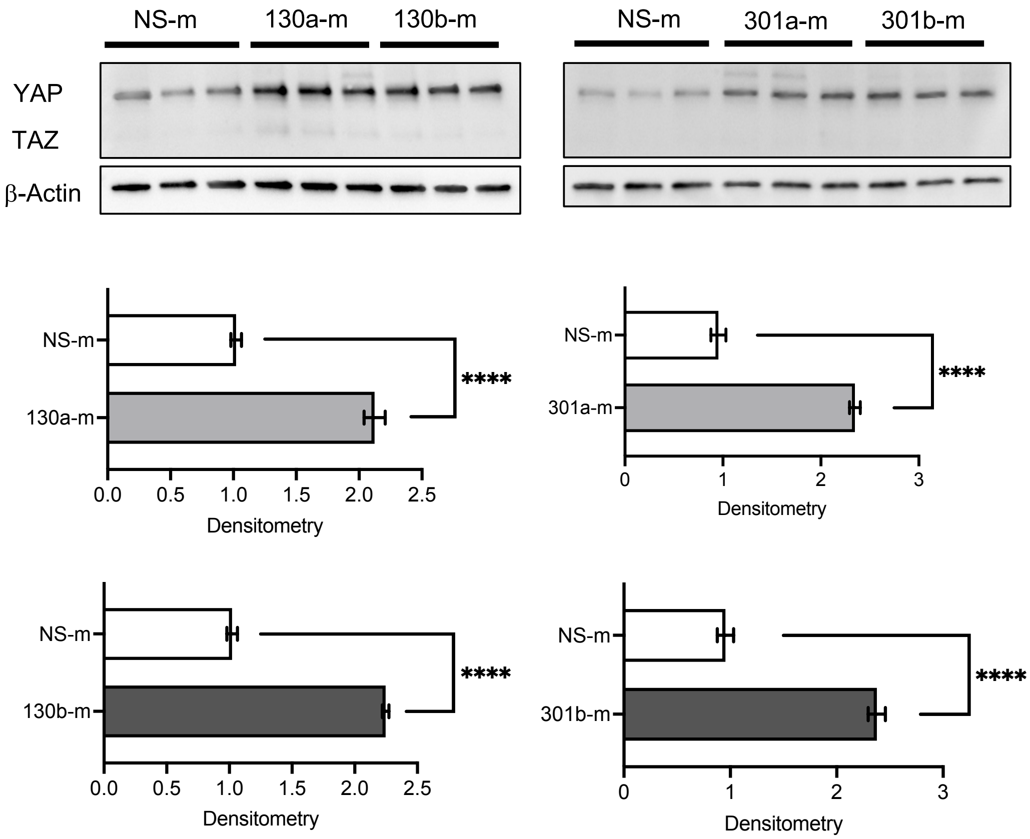

Supplementary Figure 9. Overexpression of miR-130/301 family members increase YAP/TAZ expression in ECs. Protein abundance of YAP/TAZ in HUVECs overexpressing *miR-130a*, *miR-130b*, *miR-301a*, or *miR-301b*. Densitometry normalized to β-Actin (n=3). All statistics performed with unpaired 2-tailed student t-test. ****p < 0.0001.

Supplementary Table 1. Human subject characteristics

Characteristic	T2D FI/II (n=7)	T2D FIII/IV (n=6)
Median Age	64.46 (56-70)	63.69 (55-72)
Race	Caucasian	Caucasian
Sex	Male	Male

Supplementary Table 2. Primers used for RT-qPCR (h=human, m=mouse)

Gene	Forward primer（5’ to 3’）	Reverse primer（5’ to 3’）
h-INHBA	CCTCCCAAAGGATGTACCCAA	CTCTATCTCCACATACCCGTTCT
h-THOP1	ATCTCCGTGCTCTGTGGTAAA	CGCTTGGTCTGCTCGATGAG
h-ADAM9	TCCATTGCTCTTAGCGACTGT	GGGGTTCAATCCCATAACTCG
h-FOSL1	CAGGCGGAGACTGACAAACTG	TCCTTCCGGGATTTTGCAGAT
h-TFB1M	GTTGCCCACGATTCGAGAAAT	GCCCACTTCGTAAACATAAGCAT
h-RPS6KA5	CAACAATCGTTCAAAAGGCCAA	CGACTGCCTAATGTGTTCCAG
h-SMARCD2	GATCCATTCCGAAAACGCCTG	TGAGGTAGAACCTTATCTGCCA
m-Inhba	TGAGAGGATTTCTGTTGGCAAG	TGACATCGGGTCTCTTCTTCA
m-Gapdh	AGGTCGGTGTGAACGGATTTG	TGTAGACCATGTAGTTGAGGTCA
m-Cxcl15	CAAGGCTGGTCCATGCTCC	TGCTATCACTTCCTTTCTGTTGC
